# New Striatal Neurons in a Mouse Model of Progressive Striatal Degeneration Are Generated in both the Subventricular Zone and the Striatal Parenchyma

**DOI:** 10.1371/journal.pone.0025088

**Published:** 2011-09-30

**Authors:** Federico Luzzati, Silvia De Marchis, Rosanna Parlato, Simona Gribaudo, Günther Schütz, Aldo Fasolo, Paolo Peretto

**Affiliations:** 1 Department of Animal and Human Biology, University of Turin, Torino, Italy; 2 Scientific Institute of the Cavalieri-Ottolenghi Foundation, Orbassano, Italy; 3 Helmholtz Professorship Molecular Biology of the Cell I, German Cancer Research Center (DKFZ), Heidelberg, Germany; University of South Florida, United States of America

## Abstract

Acute striatal lesions increase proliferation in the subventricular zone (SVZ) and induce migration of SVZ neuroblasts to the striatum. However, the potential of these cells to replace acutely degenerated neurons is controversial. The possible contribution of parenchymal progenitors to striatal lesion-induced neurogenesis has been poorly explored. Here, we present a detailed investigation of neurogenesis in the striatum of a mouse model showing slow progressive neurodegeneration of striatal neurons, the *Creb1^Camkcre4^Crem^−/−^* mutant mice (CBCM). By using BrdU time course analyses, intraventricular injections of a cell tracker and 3D reconstructions we showed that neurodegeneration in CBCM mice stimulates the migration of SVZ neuroblasts to the striatum without altering SVZ proliferation. SVZ-neuroblasts migrate as chains through the callosal striatal border and then enter within the striatal parenchyma as individual cells. In addition, a population of clustered neuroblasts showing high turnover rates were observed in the mutant striatum that had not migrated from the SVZ. Clustered neuroblasts might originate within the striatum itself because they are specifically associated with parenchymal proliferating cells showing features of intermediate neuronal progenitors such as clustering, expression of EGF receptor and multiple glial (SOX2, SOX9, BLBP) and neuronal (Dlx, Sp8, and to some extent DCX) markers. Newborn striatal neurons had a short lifespan and did not replace projection neurons nor expressed sets of transcription factors involved in their specification. The differentiation failure of endogenous neuroblasts likely occurred cell autonomously because transplanted wild type embryonic precursors correctly differentiated into striatal projection neurons. Thus, we propose that under progressive degeneration, neither SVZ derived nor intra-striatal generated neurons have the potential to differentiate into striatal projection neurons.

## Introduction

In rodents, the subventricular zone (SVZ)- olfactory bulb (OB) system and the dentate gyrus (DG) of the hippocampus are the major sites of adult constitutive neurogenesis under physiological conditions [1]. Different brain lesion paradigms can further induce neurogenic events in normally non-neurogenic sites, such as the CA1 of the hippocampus, the striatum or the neocortex [2]–[7]. It is known that after a lesion, neural progenitors in both the SVZ and DG increase their proliferation, and that SVZ neuroblasts migrate toward injured areas [7]. The possible contribution of parenchymal progenitors to lesion-induced neurogenesis has been poorly explored. However, recent data indicate that mild ischemia can induce genesis of cortical interneurons from progenitors located in the rat neocortical layer 1 [8]. Interestingly enough, parenchymal neuronal progenitors normally occur in the adult rabbit striatum [9]. Contrary to what is generally thought these studies maintain that the mature brain parenchyma can be permissive for the generation of neurons.

Newborn cells in injured regions generally show low survival rate, and although replacement of a few lost neuronal cells has been reported, the fate/functional meaning of lesion-induced neuroblasts remains unclear [2], [3], [5], [6]. In the striatum, where reactive neurogenesis has been extensively analyzed [2], [3], [6], the potential of newborn cells to replace medium spiny neurons, the main striatal cell type, is controversial [2], [9]. Indeed, lesion induced striatal neuroblasts are generated in the SVZ [9], [11] and express Sp8, a transcription factor associated to SVZ neuroblasts committed to becoming OB and striatal calretinin interneurons [9], [12]. This support the fact that acute injuries of the striatum do not alter the intrinsic differentiation potential of SVZ-derived neuroblasts [9].

It is to be noted that most data regarding lesion-induced neurogenesis in the striatum have been obtained in acute models of neurodegeneration, in which neuronal death mostly occurs synchronously [2], [3], [6], [10]–[12]. By contrast, only a few data are available in models of slow progressive neurodegeneration [13]. In such a state, factors modulating newborn neuron production, migration and survival, such as inflammation and astrogliosis, are expected to differ in respect to acute injuries [7], [14], [15].

Here, we present the first detailed investigation of induced neurogenesis in the striatum of a mouse model of slow progressive neurodegeneration, the *Creb1^Camkcre4^Crem^−/−^* double mutant mice, characterized by genetic ablation of the cAMP-dependent transcription factors CREB and CREM (CBCM; [16]]. In these animals, the loss of Creb1 in a Crem null background, is conditionally restricted to CamKIIα expressing mature neurons. This leads to a slow progressive degeneration of the DG, CA1 and dorso-lateral striatum [16]. Interestingly, impairment of CREB mediated transcription has been proposed as a possible cause of cell death in Huntington disease (HD). [17], [18]. In these mice we investigated both the origin and fate of newborn striatal neurons. Our data indicate that similarly to acute lesions, neurogenesis occurs within the striatum of CBCM mice, although in absence of proliferation induction in the SVZ. Notably, in parallel to migration of SVZ-neuroblasts to the mutant striatum, we found a population of clustered neuroblasts generated from intrastriatal proliferating cells showing features of intermediate neuronal progenitors such as clustering and co-expression of peculiar glial and neuronal markers [1]. However, as in acute models of neurodegeneration, newborn neuronal cells in CBCM striatum showed transient existence and did not express any specific markers of either mature or immature striatal projection neurons.

## Materials and Methods

### Animals, 5-bromo-2-deoxyuridine injections, and tissue preparation

All experimental procedures were in accordance with the European Communities Council Directive of 24 November 1986 (86 609 EEC), the Italian law for the care and use of experimental animals (DL116 92) and approved by the Italian Ministry of Health and the Bioethical Committee of the University of Turin. All experiments were designed to minimize the numbers of animals used and their discomfort.

Animal experiments were performed on male and female mice with targeted deletion of the *Creb1* gene achieved by the Cre-loxP system. *Creb1^lox^* ^P*/lox*P^ mice in which the exon carrying CREB1 DNA binding domain is flanked by loxP sites were crossed with the transgenic line carrying the Cre recombinase expressed under the control of the *CamkIIα* promoter and with mutants containing a germline deletion of the *Crem* gene [16]. Since *Crem^−/−^* male mice are sterile, we used the following mating schemes to obtain experimental mutants: (i) *Camkcre4 Creb1^lox^* ^P*/lox*P^ *Crem* ^+/−^ males×*Creb1^lox^* ^P*/lox*P^ *Crem^−/−^* females (25% probability of obtaining a double mutant; CBCM).

Animals not expressing the Cre were used as controls. Twenty eight mice aged six to nine months, 14 CBCM mutants and 14 controls, were intraperitoneally injected with 5-bromo-2-deoxyuridine (BrdU; Sigma, Steinheim, Germany; 40 mg/kg body weight in 0.1 M Tris) and than killed 2 h after a single BrdU injection (n = 4), or 5 (n = 3), 15 (n = 4) and 28 days (n = 3) after the first of 5 days of one daily BrdU injection. Animals were deeply anesthetized with a ketamine/xylazine solution (100 and 33 mg/kg body weight, respectively) and transcardially perfused with ice-cold saline solution (0.9% NaCl), followed by a freshly prepared solution of 4% paraformaldehyde (PFA) plus 2% picric acid in 0.1 M sodium phosphate buffer, pH7.4 Brains were then postfixed overnight, cryoprotected, frozen at −80°C, and sectioned in series on a cryostat. Most brains were cut into 40 µm thick sections, with the exception of six hemispheres that were used for the phenotypic analysis of BrdU+ cells at 15 d and 28 d, which were cut into 25 µm thick sections, and the Cell tracker green injected brains that were cut into 60 µm thick sections.

### Stereotaxic Surgery and Cell Tracker Green (CTG) Injections

Seven month-old adult CBCM mice were anesthetized by intraperitoneal injection of ketamine (100 mg/kg; Ketavet; Bayer, Leverkusen, Germany) supplemented by xylazine (5 mg/kg; Rompun; Bayer) and positioned in a stereotaxic apparatus (Stoelting, Wood Dale, IL). The skull was exposed by a skin incision and small holes drilled through the skull and 1 µl of Cell Tracker Green CMFDA (10 m*M* in dimethylsulfoxide; Molecular Probes, Eugene, OR, Cat #2925) was bilaterally injected at stereotaxic coordinates of 0.3 mm AP, +/− 1 mm ML, and −1.5 mm DV, using a glass micropipette and a pneumatic pressure injection apparatus (Picospritzer II, General Valve Corp, Fairfield, IL). Infusions were performed in 10–15 minutes and the micropipette was left in place for a further 10 minutes. After the micropipette was removed, the skin was sutured with 8.0 mm silk thread. Following surgery, the animals were sacrificed at 24 hr (n = 3) or seven days (n = 3) following surgery.).

### Preparation of cell suspension and transplantation

For embryonic precursor transplantation, donor cells were dissected from embryo [embryonic day 15 (E15) EGFP mice. The preparation of donor Lateral Ganglionic Eeminence (LGE) progenitor cells was performed as described previously [19], [20]. Briefly, the LGE of donor embryos was isolated and mechanically dissociated to a single-cell suspension in ice-cold Leibovitz L15 medium. The obtained suspension was centrifuged and resuspended in the same solution. The donor cell suspensions were re-suspended at a final concentration of 5×10^4^ cells/µl.

Cell transplantation was performed on CBCM (n = 3) and control mice (n = 3), following the same surgical procedure described previously for neuronal tracer injection. In all experiments, 1.5 µl of the cell suspension were bilaterally injected at +1. mm AP, +/− 2. mm ML, and 2.5 mm DV to reach the dorso-lateral left striatum. Infusions were performed in 10–15 minutes and the micropipette was left in place for a further 10 minutes. Animals were left to survive for 21 days after surgery.

### Immunohistochemistry

Immunohistochemical reactions were performed on sections incubated for 24–48 h at 4°C in a solution of 0.01 M PBS, pH 7.4, containing 0.5–1% Triton X-100, normal serum and the following antibodies: ***Markers of Proliferation***: anti-BrdU, 1∶3000 (rat monoclonal, AbD Serotec, Kidlington, UK); anti-Ki67, 1∶500 (mouse monoclonal, BD Pharmingen, San Diego, CA); anti-Ki67 1∶1000 (rabbit polyclonal; Novocastra, Benton Lane, UK); ***Markers of the neuronal lineage***: anti-class III b-tubulin, 1∶600 (TU-J1, mouse monoclonal and polyclonal; Babco, Richmond, CA); anti-neuronal-specific nuclear protein (NeuN), 1∶1000 (mouse monoclonal; Chemicon, Temecula, CA); anti-doublecortin (DCX), 1∶500 (goat polyclonal sc-8066; Santa Cruz Biotechnology, Santa Cruz, CA); anti-doublecortin (DCX), 1∶1000 (rabbit polyclonal, Cell signalling, Beverly, MA); anti-PSA-NCAM, 1∶4000 (monoclonal IgM; G. Rougon, Marseille, France); anti pan-DLX 1∶1000 (rabbit polyclonal; [53] ***Markers of the glial lineage:*** anti- SOX10, 1∶1000 (goat polyclonal sc-17342; Santa Cruz Biotechnology, Santa Cruz, CA); anti- SOX2, 1∶1000 (rabbit polyclonal, Chemicon, Temecula, CA); anti-SOX9, 1∶1000 (rabbit polyclonal, Millipore, Billerica, MA); anti-brain lipid binding protein (BLBP), 1∶800 (polyclonal; Todd Anthony, Rockefeller University, NewYork, NY).

***Transcription factors involved in neuronal subtype specification:*** anti-Lhx6, 1∶1000 (rabbit polyclonal; (Grigoriou et al., 1998); anti-Nkx2.1, 1∶1000 (Biopat s.r.l., Piedimonte Matese, Italy); anti-PAX6 1∶1000 (Rabbit polyclonal, Chemicon, Temecula, CA); anti-CTIP2 1∶1000, anti-Sp8 1∶10000 (rabbit polyclonal, Millipore, Billerica, MA), anti-Isl1 1∶1000 (clone 39.4D5, mouse monoclonal, DSHB, Iowa City, Iowa), anti-FOXP2 1∶1000 (rabbit polyclonal, Abcam, Cambridge, UK); anti-Lhx6, 1∶1000 (rabbit polyclonal; [54]).

***Markers of striatal neurons:*** anti-parvalbumin, 1∶2000 (rabbit polyclonal; Swant, Bellinzona, Switzerland); and anti-somatostatin, 1∶2000 (a-SRIF, rabbit polyclonal; a kind gift from Dr R. Benoit, Montreal, Quebec, Canada); anti-calretinin, 1∶2000 (rabbit polyclonal; Swant), anti-dopamine and cAMP-regulated phosphoprotein (DARPP-32), 1∶1000 (monoclonal; BD Transduction Laboratories, Lexington, KY); anticholine acetyltransferase, 1∶1000 (goat polyclonal; Chemicon Temecula, CA);, anti-tyrosine hydroxylase (TH) 1∶2000 (rabbit polyclonal; Institut Jacques Boy, Reims, France).

#### Other Markers

In addition, we used an anti-GFP 1∶1000 (chicken polyclonal; Aveslab, Tigard, Oregon); anti-Cre recombinase 1∶3000 (rabbit polyclonal, Covance Research Products, Princeton, New Jersey); anti-EGFr 1∶200 (sheep polyclonal; Upstate biotechnology, Lake Placid, NY).

For BrdU staining, DNA was denatured in 2N HCl for 30 min at 37°C. Sections were then rinsed in 0.1 M borate buffer, pH 8.5. Following primary antisera incubation, sections were incubated with appropriate solutions of secondary cyanine 3 (Cy3) -conjugated (1∶800; Jackson ImmunoResearch, West Grove, PA), Alexa488-conjugated (1∶200; molecular probes) and dylight 649-conjugated (1∶100 donkey, Jackson ImmunoResearch, West Grove, PA) antibodies. For GFP staining we used a FITC conjugated anti-chicken (1∶100, goat polyclonal; Aveslab, Tigard, Oregon). For simultaneous labelling of four antigens we also used a biotinylated a-Rat (1∶150; Vector Laboratories, Burlingame, CA) followed by incubation with AMCA-avidinD (1∶100; Vector Laboratories, Burlingame, CA). Sections were then coverslipped with antifade mounting medium Dabco (Sigma) and analyzed with a laser scanning Olympus Optical (Milan, Italy) Fluoview confocal system (Olympus Optical), with a laser scanning Leica TCS SP5 (Leica Microsystems) or with a Nikon 80i associated with the software Neurolucida (MicroBrightField, Colchester, VT). Images were processed using NIH image J (http://rsb.info.nih.gov/ij/) and Adobe Photoshop 7.0 (Adobe Systems, San Jose, CA) and assembled into montages using CorelDraw 11 (Corel, Ottawa, Ontario, Canada). Only general adjustments to color, contrast, and brightness were made.

### Light and confocal 3D reconstructions

For 3D reconstructions, one hemisphere of a CBCM mouse and one of a control mice were serially coronally sectioned (40 µm thick) and immunolabeled with anti-DCX. For the reconstruction of the SVZ, dPSB and striatal chains along the entire brain, 140 and 120 consecutive sections, from control and CBCM mice respectively, encompassing the entire SVZ and lateral ventricle were digitally captured (voxel size 0.7×0.7×40 µm) and reciprocally aligned using Reconstruct software version 1.1 (Copyright 2004, John C. Fiala, Boston University, Boston, MA; http://tech.groups.yahoo.com/group/reconstruct_users). The dPSB chains, CC DCX+ cells and striatal DCX+ clusters were 3D rendered as *traces slabs*, in which *traces* were filled with colour and extruded along the z-axis, while the SVZ was rendered as *Boissonnant surface*, in which subsequent traces were interpolated. For higher resolution 3D reconstructions subsequent sections were acquired by the confocal microscope at 20× (N.A. 0.7; size 0.7×0.7×2 µm), or 100× (N.A. 1.3; 0.13×0.13×1 µm). Multiple stacks from the same section were mounted with Vias (CNIC, University of New South Wales, Australia) and the sequences of optical planes from subsequent sections were imported in Reconstruct for alignment. Tracings of 20× reconstructions were obtained in reconstruct using the wildfire region growing tool, and were represented as *traces slabs* and rendered in Blender (Stichting Blender Foundation, Amsterdam, Netherlands; www.blender.org). Tracings of the blue and cyan groups shown in supplementary Video S1 were obtained through an automatic algorithm in Neuronstudio ([21]; CNIC, University of New South Wales, Australia) and were rendered in V3D neuron ([22]; http://penglab.janelia.org/proj/v3d). Single DCX+ cells were traced and analyzed with a semi-automatic procedure in Neuromantic 1.6 (Darren Myatt, University of Reading, UK).

### Quantifications and statistical analyses

*Volumes* were evaluated with Neurolucida 7.0 in a 1-in-6 series of sections (40 µm thick) labeled with DAPI from CBCM (n = 3) and control mice (n = 3). *Total number of cells* was evaluated stereologically with Neurolucida in a 1-in-6 series of sections (40 µm thick) in both control (n = 3) and CBCM (n = 4) mice. In the present study the optical fractionator principle was modified, in that, only the labelled cells in the uppermost focal plane were excluded to avoid oversampling, as described by others [23], [24].

The percentage of Ki67+/DCX+ cells (K/D+) and Ki67+ cells associated with DCX+ cells (K/aD cells) labeled for SOX2, SOX9, BLBP, EGFr, pan-Dll and BrdU was evaluated in at least 80 cells per animal for each cell type.

*Analysis of the maturation of newly generated neuronal precursors in the striatum of CBCM mice.* This analysis was performed on sections labelled simultaneously for BrdU, DCX, NeuN and CaMKII-Cre in CBCM mice at 15days (n = 3) and 28 days (n = 3) from BrdU administration. Total number of BrdU+ cells in the striatum was counted stereologically in Neurolucida. The proportion of cells co-labeled for BrdU and combinations of DCX, NeuN and CaMKII-Cre compared to the total BrdU+ population was calculated by sampling 100 BrdU positive cells per animal. This proportion was then multiplied for the total number of BrdU+ cells to obtain estimates of the total number of each specific subpopulation of BrdU+ cells.

*Statistical analyses* were performed by using Statistical Package for the Social Science 14.0 (SPSS, Chicago, IL). Analysis of variance (ANOVA) was followed by Tukey's *post hoc* test when appropriate.

## Results

### The dorsal SVZ expands laterally in CBCM mice

**Figure 1:**
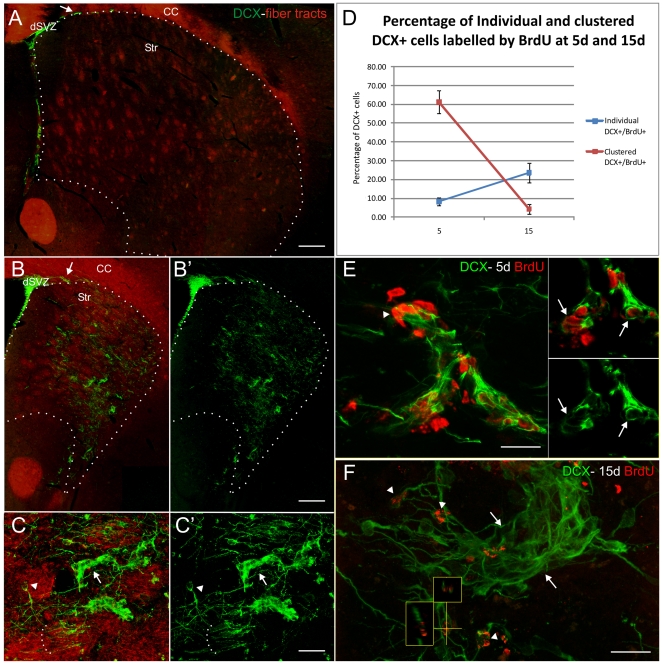
Neurogenic response to neurodegeneration in the CBCM mice brain. **A–C′**) Coronal sections cut at the level of the caudal nucleus accumbens of control (A) and CBCM mice (B,C) labeled for DCX (green). In A,B and C, DCX staining was merged with a bright-field acquisition inverted and pseudocolored in red to show fibre tracts. DCX+ cells are more abundant than in controls along the border between the corpus callosum and the striatum (arrow in B′). In A, B and B′ the striatal border is indicated by a dotted line. **C–C′**) The DCX+ cells in the striatal parenchyma are organized as clusters (arrow) or as single cells (arrowhead). The latter cells are often fascicled (dotted line). **D**) Percentage of individual DCX+ cells (blue) and clustered DCX+ cells (red) that are BrdU labeled in the CBCM striatum at 5 and 15 days after the first of 5 BrdU injections (1 injection per day). **E–F**) BrdU + nuclei (red) and DCX positive cells (green) in the ventro-lateral caudate putamen of CBCM mice 5 (E) and 15 (F) days after the first of 5 BrdU injections (1 injection per day). In the insets in E a single confocal planes showing the colocalization of BrdU and DCX in clustered DCX+ cells (arrow). In F, the BrdU+ cells at 15 d are mostly found among individual DCX+ cells (arrowheads). Small panels show orthogonal views of a BrdU/DCX+ cell along the yellow lines. A DCX+ cluster is indicated by two arrows. In E, note the occurrence of BrdU positive and DCX negative nuclei closely associated with the cluster of DCX+ cells (arrowhead). Abbreviations: dSVZ: dorsal Subventricular zone; Str: striatum; CC, corpus callosum. Ref bars: 230 µm in A,B; 25 µm in C,C′; 20 µm E,F.

**Figure 2:**
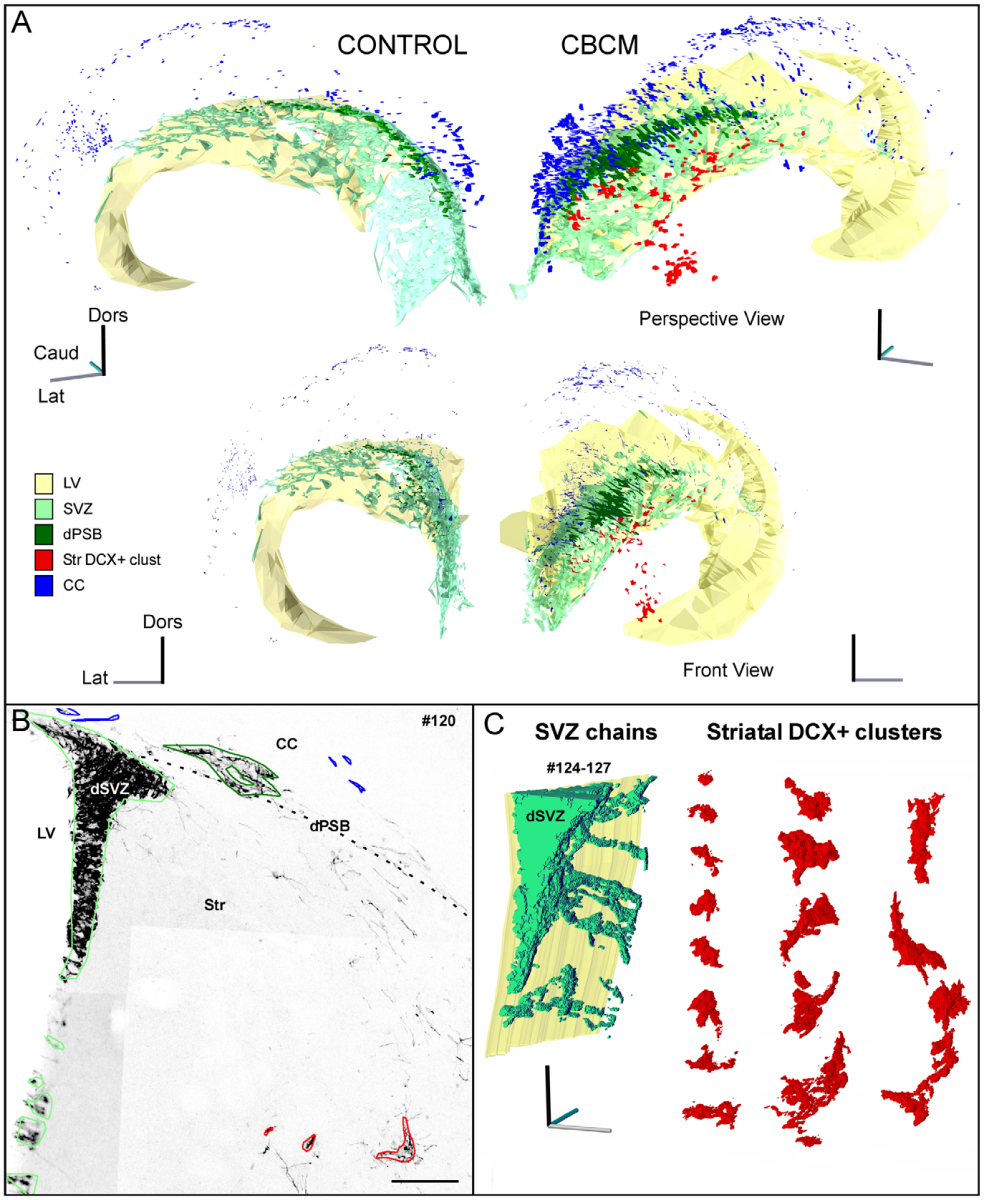
3D reconstruction study of the striatal DCX+ clusters and their relationships with the SVZ and dPSB in control and mutant animals. **A**) 3D models obtained from serial section reconstruction of 40 µm thick coronal sections taken out of control (on the left) and CBCM (on the right) mice (voxel size: 0,7×0,7×40 µm). Perspective (on top) and front views (on bottom) are shown. The X (gray), Y (dark) and Z (cyan) axes are indicated for each view. In both control and CBCM mice, DCX+ cells in the SVZ (green) constitute a network on the surface of the LV (yellow). Neuroblasts in the dPSB (dark green) and striatal DCX+ clusters (red) strongly increase in the CBCM mice. Note that DCX+ clusters in the striatum are not organized in a network. **B**) Single coronal section from the CBCM reconstruction in which diverse structures are outlined with different colours, according to the legend in A. **C**) Reconstructions at higher resolution (voxel size 0,7×0,7×2 µm) of dorsal SVZ chains included in three consecutive sections (#124-127; green) and striatal DCX+ clusters taken from the reconstruction shown in Fig. 3B (red). The ependymal surface is shown in yellow. SVZ chains are elongated and coalesce in the dSVZ. By contrast, DCX+ clusters (red) have irregular shapes, and lack any preferred orientation. Scale Bars 100 µm in B.

**Figure 3:**
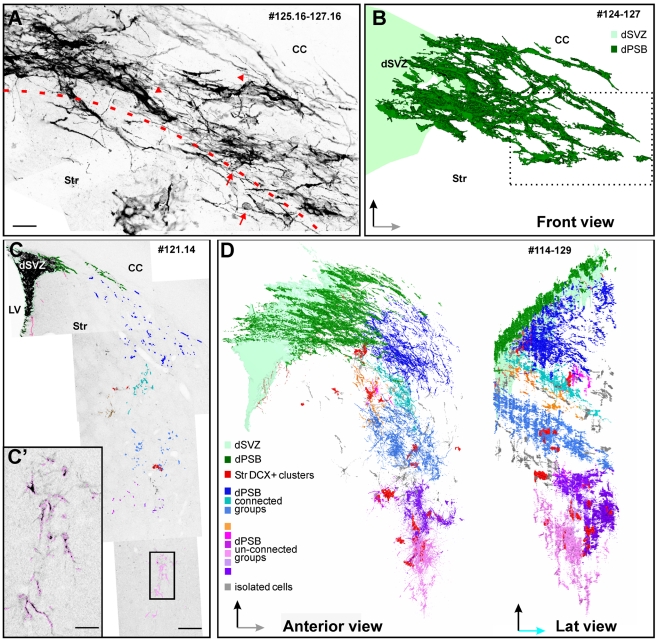
3D reconstruction study: analysis of induced DCX+ cells in the dSVZ, dPSB and striatum of CBCM mice. **A**) Z projection of 80 confocal planes taken at the level of the lateral dPSB obtained from three subsequent coronal sections (#125.16–127.16). The dPSB chains (arrowheads) are surrounded by several individual cells (arrows). The callosal-striatal border is indicated by a dotted line. **B**) 3D reconstruction (voxel size 0,13×0,13×1 µm) of dPSB chains (dark green; slices #124–127). Note that these chains organize in a network originating from the dSVZ (light green). The dotted line indicates the location of the image depicted in Fig. 3A. **C-C′**) A single confocal plane (#.14) of a DCX stained coronal section (#121) from the reconstruction shown in D. Outline colors are as legend in D. In **C**′ a higher magnification of the panel in C. **D**) Anterior (left) and lateral (right) views of the 3D models obtained from the outlines traced on 320 optical planes taken from 16 subsequent sections 40 µm thick (voxel size 0,7×0,7×2 µm; sections #114–119 of the CBCM animal are shown in Fig. 2. In the reconstructed volume DCX+ clusters (red), the dPSB (dark green) and the dSVZ (pale green) are shown together with nine independent groups of individual DCX+ cells. Three of them contact the dPSB (*dPSB-connected groups*; blue tones) while the other six do not (*dPSB-unconnected groups*;orange, and pink/violet tones). The contact between the dark cyan group and the dPSB occurs anteriorly to the reconstructed volume. Scale bars 100 µm in A, 23 µm in A′, 10 µm in C.

In CBCM mice the loss of Creb1 under the control of CamKIIa promoter, in absence of Crem expression, leads to progressive neurodegeneration of the dorsolateral striatum [16]. According to previous studies [16], the volume of the caudate/putamen of six month old CBCM mutants was reduced about 70% compared to control mice (Volume of the Striatum: Controls = 6.5±1.1 mm^3^; Mutants = 2.2±0.3 mm^3^; F_2,1_ = 43.83 p = 0.003; n = 3; Fig. 1A,B) while the volume of the lateral ventricle was about 250% expanded (Volume of the lateral ventricle: Controls = 0.99±0.30 mm^3^; Mutants = 2.47±0.62 mm^3^; F_2,1_ = 13.69 p = 0.021; n = 3). In acute models of striatal neurodegeneration the SVZ increases its proliferative activity and acts as a source of new neurons for the striatum [2], [6], [11], [25]. In CBCM mice, despite a severe striatal neurodegeneration the SVZ volume and the number of proliferating cells labeled by a two hour BrdU pulse in this region were comparable to those of controls (Volume of the SVZ: CBCM = 0.060+/−0.004 mm^3^; Controls = 0.044+/−0.011 mm^3^, F_2,1_ = 5.8, p = 0.07; BrdU+ cells: CBCM 5344+/−894; controls 5656+/−2092 cells; p = 0.677; n = 3 animal/group). Nevertheless, doublecortin (DCX) immunostaining in mutants revealed an expansion of the dorsal portion of the SVZ (dSVZ, Fig. 1A,B, Fig. 2B; Volume of the dSVZ: CBCM = 0.0049+/−0.0009 mm^3^; Controls = 0.0251 +/−0.0084 mm^3^, F_2,1_ = 17.3, p = 0.014). A net increase in DCX+ cells was also found along the boundary between the corpus callosum and the striatum, here referred to as the dorsal pallial-subpallial boundary (dPSB; arrow in Fig. 1A,B; dark green in Fig. 2A,B, Fig. S1). As shown by a 3D reconstruction study, neuroblasts in the dPSB formed laterally oriented chains organized in a network directly linked to the dSVZ (Fig. 3B). In addition, many individual DCX+ cells were observed along the striatal side of the dPSB (Fig. 3A, arrows), suggesting the occurrence of SVZ neuroblast migration through the dPSB to the striatum. Thus, in CBCM mice the lateral extension of the SVZ along the dPSB might represent a specific migratory pathway leading neuroblasts towards the dorso-lateral striatum.

### The caudate-putamen of CBCM mice is enriched by newly generated DCX+ cells

In the mutant striatum numerous DCX+ cells were identified (Fig. 1B–C). These cells expressed the neuronal marker beta-III tubulin, but not the glial markers GFAP, SOX10 or RIP (data not shown) indicating they were in the neuronal lineage. The number of DCX+ cells in the caudate-putamen was about 100 times higher in mutants compared to controls (CBCM 29280+/−6504; controls 216+/−192; F_1,2_ = 59.85; p = 0.016; n = 3 animals/group). DCX+ cells were particularly enriched in the dorso-lateral caudate-putamen (Fig. 1B,B′), which corresponds to the striatal sub-region most affected by neuronal degeneration [16]. In mutants, most striatal DCX+ neuroblasts were organized as individual cells (41.9+/−3.8%; arrowheads in Fig. 1C,C′) or as clusters of at least four cells with tightly packed cell bodies (51.2+/−4.0%; arrows in Fig. 1C,C′). Smaller DCX+ cell clusters (2–3 cells) were rarely observed (6.9+/−0.6%).

In order to investigate whether striatal DCX+ cells were newly generated, BrdU was given for 5 consecutive days (1 injection/day; 50 mg/Kg) and the animals were left to survive for 5 (5 d; n = 3) and 15 days (15 d; n = 3) from the first injection. At 5 d, 61.2+/−6.1% of the clustered DCX+ cells were labeled by BrdU, whereas only 8.3+/−2.1% of the individual DCX+ cells were BrdU+ (Fig. 1D, E). At 15 d the percentage of BrdU labeled cells dropped to 4,4+/−2,5% for clustered DCX+ cells (F_1,2_ = 221.6; p = 0.001), while it raised to 23.6+/−5.1% for individual DCX+ cells (F_1,2_ = 22.4; p = 0.02;Fig. 1D,F). These data indicate that newborn striatal DCX+ cells are initially organized mostly in clusters.

### Morphology and spatial distribution of striatal DCX+ neuroblasts

#### Clustered DCX+ cells

DCX+ clusters in mutant striatum showed a stereotyped distribution, typically occupying a dorsal position in the anterior striatum, a central and ventro-lateral position in the middle striatum and a medial position in the caudal striatum (Fig. 2A; Fig. S1). Clusters were restricted to the grey matter (Fig. 1C) and generally showed irregular profiles, lacking a preferential orientation (Fig. 2C). A 3D reconstruction showed that unlike SVZ and dPSB chains, striatal DCX+ clusters were scattered and not organized as a network (Fig. 2A,C; Fig. S1). None of the clusters directly come in contact with the SVZ or the dPSB.

#### Individual DCX+ cells

The spatial distribution of striatal individual DCX+ cells overlapped that of clustered cells. In addition, individual DCX+ cells were also found in the lateral sub-callosal region (Fig. 3C,D). In contrast to the clustered cells, individual cells were found both in the grey matter and within the fiber tracts of the internal capsule where they were often organized in fascicles (Fig. 1C dotted line).

**Figure 4:**
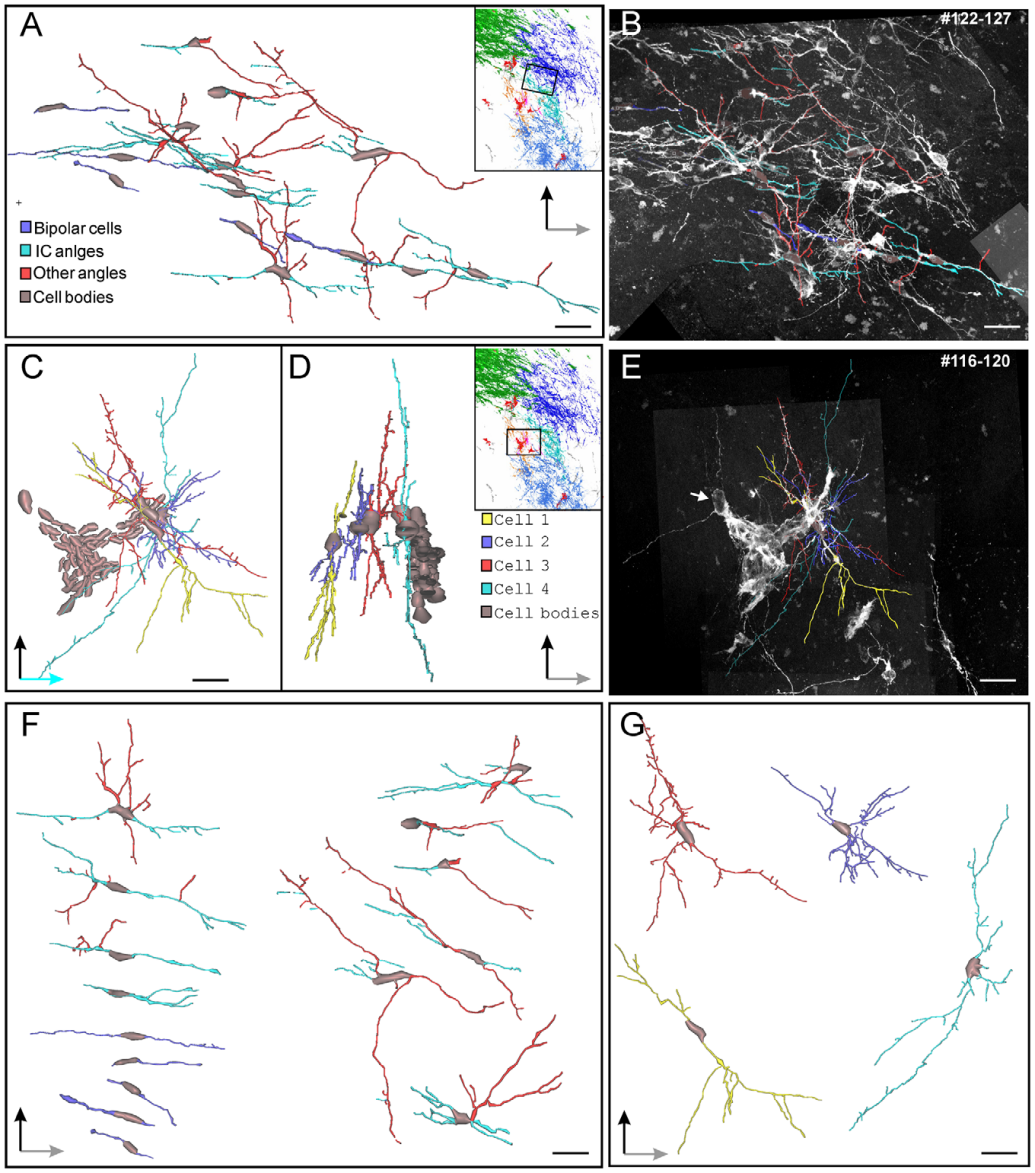
3D reconstruction study: analysis of single DCX positive cells in the striatum of CBCM mice. **A**) Front view of a 3D reconstruction (voxel size 0,13×0,13×1 µm) of 15 cells included in a tract of 240 µm of the *dPSB-connected* cyan group shown in fig. 3B. Individual views of each cell are represented in **F**. **B**) Z projection of the confocal planes from which the reconstruction shown in A has been obtained. Most cells have processes running with a slope similar to that of the internal capsule fibers bundles (IC angles, cyan: 25–35° on the medio-lateral and 20–30° on the rostro-caudal axes). Cell Processes extending in other directions are in red. Five cells showed bipolar morphology (violet). Cell bodies are in gray. **C**) Front view of a 3D reconstruction (voxel size 0,13×0,13×1 µm) of four cells (each drawn in different colour) included in a tract of 200 µm of the *dPSB-unconnected* pink group shown in fig. 3B. These cells are located close to a cluster of DCX+ cells. **D**) Side view of the reconstruction in C (the Z dimension is reduced by about half its real value). Individual views of each cell are represented in **G**. **E**) Z projection of the confocal planes from which reconstructions shown in C,D have been obtained. Scale bars: 20 µm.

To better understand the spatial organization of the individual DCX+ cells and their relationships with the SVZ and the DCX+ clusters, we performed a 3D reconstruction of the middle striatum, spanning 640 µm along the rostro-caudal axis (Fig. 3). In the reconstructed volume we identified nine spatially segregated groups of individual DCX+ cells (Fig. 3C,D blue and violet tones). None of these groups directly contacted the SVZ, while three of them did, indirectly through the dPSB (dPSB connected group, blue tones Fig. 3D). The dPSB connected groups were restricted to the dorsal caudate-putamen and extended caudo-ventrally for several hundred microns with a slope similar to that of the bundles of the internal capsule (Fig. 3D; Fig. S2). Some of the DCX+ cells in the dPSB connected groups were bipolar (blue in Fig. 4A, F), but most of them showed a more complex morphology with long neurites often oriented parallel to the internal capsule fibers (cyan in Fig. 4A, B, F). Interestingly, the dPSB connected groups were only rarely associated with the clustered DCX+ cells (red in Fig. 3D; Supplementary Video S1).

The dPSB unconnected groups were located in the medial and ventral caudate-putamen (Fig. 3C–D). Cells in these groups were only rarely oriented along the internal capsule fiber bundles (Fig. 4C–E, G; data not shown). In contrast to the dPSB connected groups, those unconnected were closely associated with the striatal DCX+ clusters (Fig. 3D).

Overall, these data indicate that a subset of individual DCX+ cells in CBCM striatum is indirectly linked with the SVZ through the dPSB. By contrast, clustered DCX+ cells and individual cells surrounding them are mostly separated from the SVZ, suggesting they are independent from this germinative region.

### SVZ-derived neuroblasts enter the striatum of CBCM mice as individual cells and do not contribute to the population of clustered DCX+ cells

**Figure 5:**
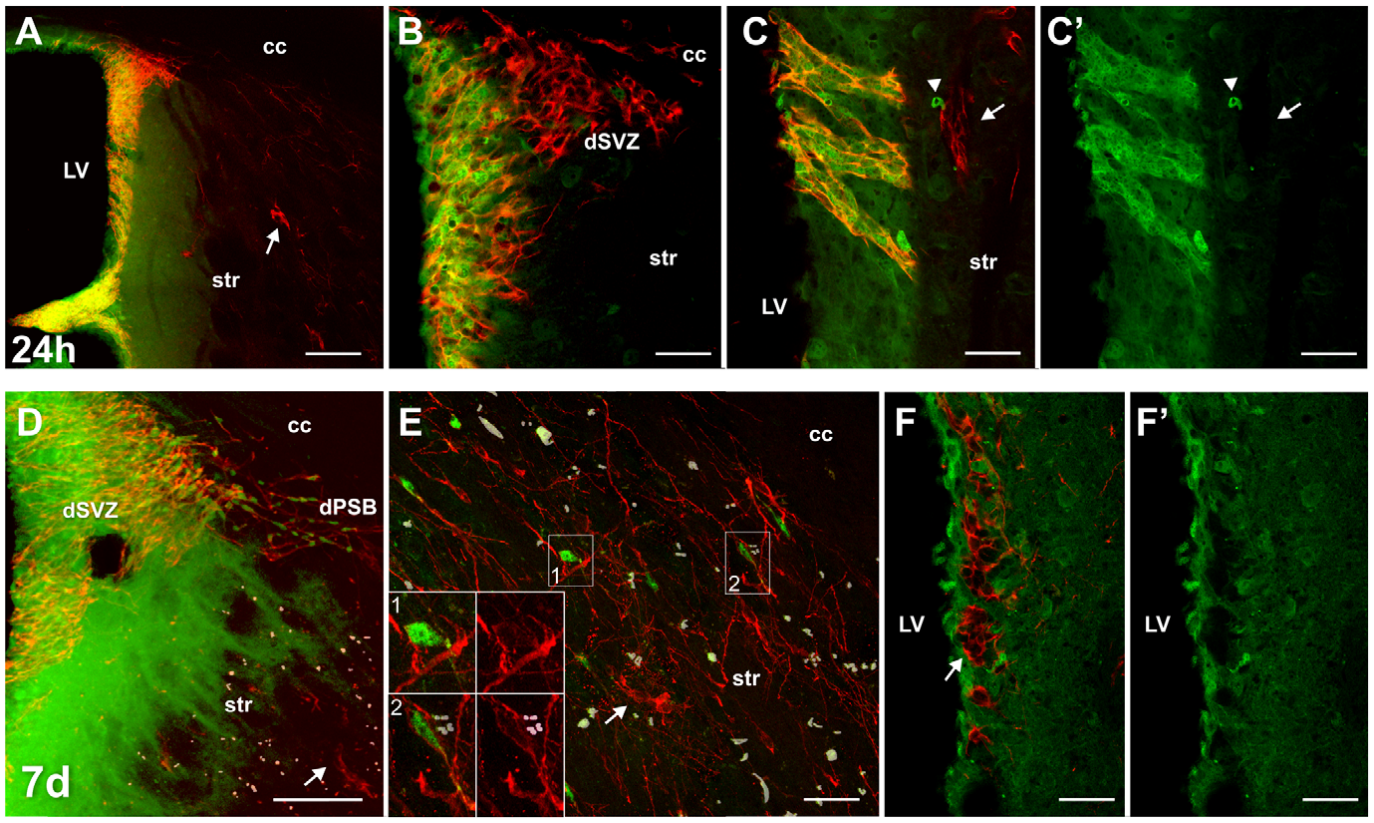
Intraventricular injections of Cell Tracker Green (CTG) in the CBCM mutants. **A–C′**) Coronal sections (60 µm thick) cut at middle (A,B) and caudal (C.C′) levels of the SVZ, at 24 hr after the tracer injection. In B a single confocal plane at higher magnification of the dSVZ from the section shown in A. The CTG+(green)/DCX+ (red) cells are restricted to the periventricular SVZ. Occasionally, some CTG+/DCX− cells are detectable in the most medial part of the striatum (arrowhead in C). Arrow in C indicate a DCX+ cluster that is not labeled by CTG. **D–F′**) Images taken from coronal sections (60 µm thick) showing the dSVZ and dPSB (D), the dorso-lateral striatum (E), and the periventricular SVZ (single confocal plane in F,F′), at 7 days after the tracer injection. Numerous CTG/DCX co-labelled cells are evident in the dSVZ, dPSB (D) and dorsolateral striatum (E). Insets in E shows higher magnification of two individual DCX+/CTG+ cells Note that all the CTG/DCX+ cells in the striatum are single cells. In D and E, the autofluorescent deposits have been colored in white to be able to avoid confusion with CTG labeling. At 7 days post CTG injection, most of the DCX+ periventricular neuroblasts (arrow in F) are not labelled by the tracer. Scale bars 100 µm in A; 50 µm in D; 25 µm in B–C′, E–F′.

In order to verify whether SVZ neuroblasts migrate into the CBCM striatum, we performed intraventricular injections of Cell Tracker Green (CTG). This cell tracker had previously been shown to efficiently label migrating SVZ neuroblasts over several days [26], [27], [9]. At 24 hours from CTG injection virtually all periventricular DCX+ cells in the SVZ of CBCM mice were labeled by CTG (Fig. 5 A–C′). By contrast, very few DCX+ cells incorporated CTG in the dSVZ, and no CTG/DCX co-labeled cells were identified in the RMS, OB, dPSB and striatal parenchyma (Fig. 5 A–C′ and data not shown). At 7 d post injection CTG+/DCX+ cells migrated out of the periventricular SVZ (Fig. 5 F–F′ and data not shown) and reached the RMS and OB (data not shown), but also the dPSB and the underlying dorso-lateral caudate-putamen (Fig. 5D,E). In the striatum CTG+/DCX+ cells were always identified as individual cells, mostly oriented parallel to the internal capsule fiber bundles (Fig. 5 D, E). No clustered DCX+ cells were observed to be CTG+ at both 24 hours and 7 d survival time (Fig. 5 D, E arrows).

These data confirm that at least a subset of individual DCX+ cells in the striatum of the CBCM mice derive from the SVZ. In parallel, they support the hypothesis that striatal DCX+ clusters do not derive from this germinative region.

### DCX+ clusters in the CBCM striatum are closely associated with intermediate progenitor-like cells

#### Analysis of the organization of proliferating cells in CBCM striatum

**Figure 6:**
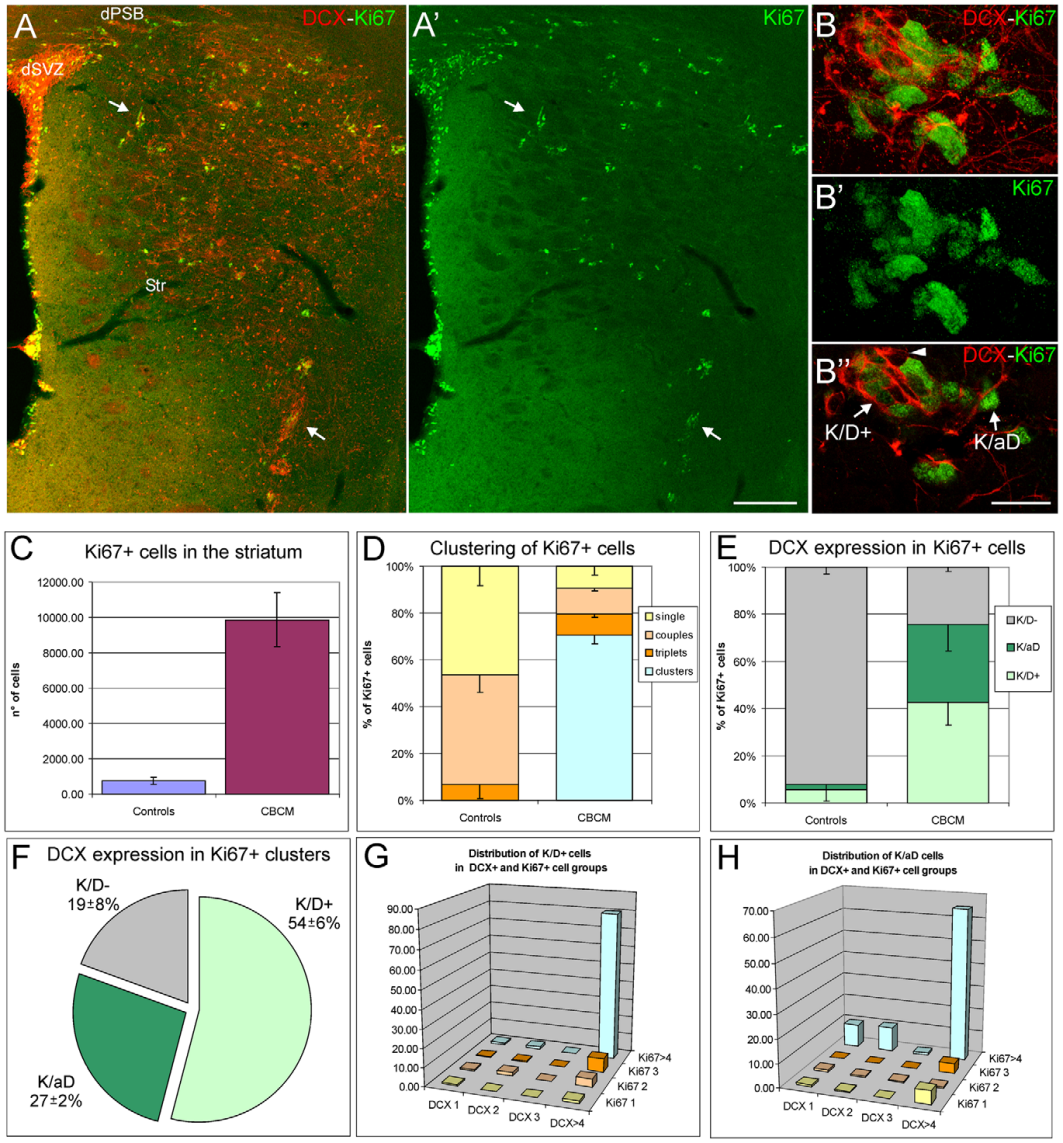
Organization of Ki67+ cells in CBCM mice striatum. **A-A′**) Immunolabelling for Ki67 (green) and DCX (red) in one coronal section cut at the level of the middle striatum. Strong association between Ki67 and DCX positive cells is visible in both SVZ and striatum (arrows A,A′). **B-B″**) One cluster of Ki67+ cells partially overlapping with a cluster of DCX+ cells. In B″ a single confocal plane of B-B′ showing K/D+ cells (Ki67+/DCX+) and K/aD cells (Ki67+/DCX− cells associated with DCX+ cells either directly or through the interposition of other Ki67+ cells). Note that some cells of the DCX+ cluster do not express Ki67 (arrowhed). **C**) Total number of Ki67+ cells in the striatum of CBCM and control mice (CBCM vs controls, p = 0.008, n = 3/group). **D**) Percentage of Ki67+ cells organized into groups of 1, 2, 3 or more than 4 cells (single couples, triplets, clusters) in the striatum of control (n = 3) and CBCM mutants (n = 3). **E**) Percentage of Ki67+ cells being isolated from DCX+ cells (K/D−); associated with DCX+ cells (K/aD); expressing DCX (K/D+) in control (n = 3) and CBCM mice (n = 3). **F**) Relative proportions of K/D−, K/aD and K/D+ cells in Ki67+ clusters of CBCM striatum (n = 3). **G, H**) 3D bar charts showing the distribution of K/D+ (G) and K/aD (H) cells in respect of groups of 1,2,3 or more than 4 DCX+ (x axis) or Ki67+ (z axis) cells. Scale bars 200 µm in A,A′; 10 µm in B-B″.

In adult neurogenic niches neuroblasts are produced by the activity of intermediate neuronal progenitors. The latter cells are in physical contact with neuroblasts and show specific features such as clustering, high proliferative activity and co-expression of glial and neuronal markers [28], [29], [30], [1]. We previously demonstrated the occurrence of intermediate neuronal progenitor-like cells in the striatum of the adult rabbit [9]. To unravel whether DCX+ clusters in the CBCM striatum could be related to the presence of local neuronal progenitors, we firstly investigated the occurrence of cells expressing the endogenous marker of proliferation, Ki67 [31], and their association with DCX+ clusters. In the CBCM striatum the number of Ki67+ cells was about ten times higher than in controls (Fig. 6 C; F_1,2_ = 107.1; p = 0.008, n = 3) and 70.6±3.8% of these cells were organized into clusters of at least four cells (Ki67+ clusters; Fig. 6A,B,D). As expected, Ki67+ clusters in control brains were exclusively found in the DG and SVZ (data not shown).

In contrast to the striatum of control mice, where only rare Ki67+ cells were labelled for DCX, in mutants 42,8±9,8% of Ki67+ cells co-expressed DCX (*K/D+*, Fig. 6B″,E). Moreover, 32,8±11% of Ki67+ cells were negative for DCX but were either in contact with DCX+ cells or were part of Ki67+ clusters contacting DCX+ cells (we will refer to these cells as Ki67+ cells associated with DCX or *K/aD*; Fig. 6B″,E). Thus, in the striatum of CBCM mice, Ki67+ cells are mostly clustered and closely associated with DCX+ cells. The analysis of the association of K/D+ and K/aD cells with individual or clustered Ki67+ and DCX+ cells (Fig. 6G,H), revealed that 80,1±10,0% of the K/D+ cells were simultaneously part of Ki67+ and DCX+ clusters (Fig. 6G), while 65,2±8,8% of K/aD cells belong to Ki67+ clusters contacting DCX+ clusters (Fig. 6H). These data indicate that the Ki67+ clusters are mainly associated, and partly overlapped with the DCX+ clusters. In particular, the K/D+ cells accounted for 53,9±6,1% of the cells of the Ki67+ clusters (Fig. 6F) and for 25,0±3,1% of the cells of the DCX+ clusters.

**Figure 7:**
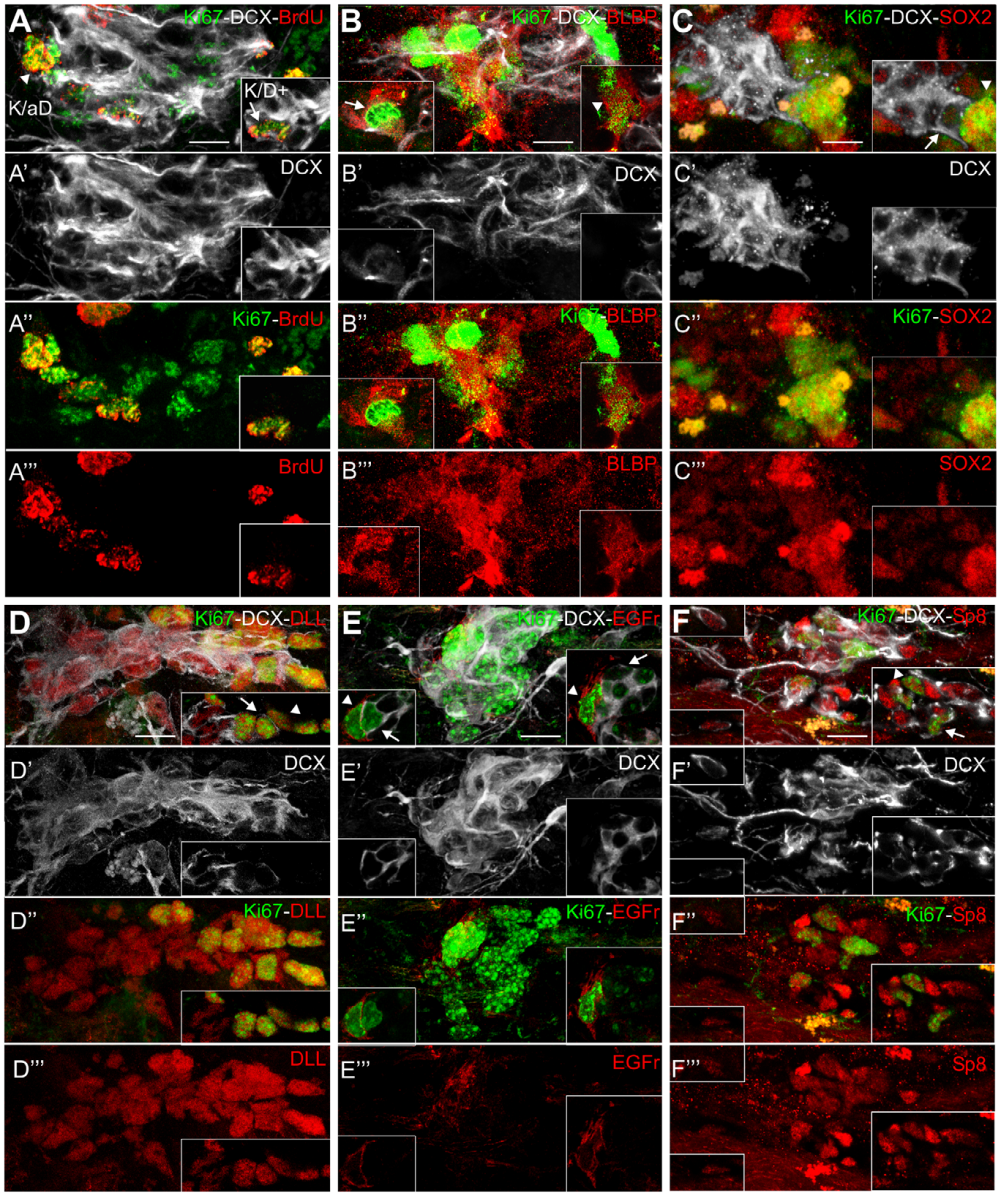
Phenotypic analysis of K/aD, K/D+ and postmitotic DCX+ cells in the striatum of CBCM mice. **A–F**) Z projection of confocal stacks taken out of 25 µm thick sections triple labelled for Ki67 (green), DCX (white) and in red: BrdU (A-A‴), BLBP (B-B‴), SOX2 (C-C‴), pan-Dll (D-D‴), EGF-receptor (E-E‴), Sp8 (F-F‴). Insets are single confocal planes in which the labelling of K/D+ (arrows), K/aD (arrowheads) and postmitotic DCX+ cells can be better appreciated. Both K/aD and K/D+ cells are labelled for BrdU, BLBP, SOX2, DLL and Sp8. In the insets in E two different K/aD cells expressing the EGFr are associated with EGFr negative K/D+ cells. In the inset in B, note that in the imaged K/D+ cell, the high Ki67 intensity and the staining pattern, are characteristic of the mitotic phase of the cell cycle (Schlozen and Gerdes 2000). Scale Bars: 10 µm in A–F‴.

A high proportion of both K/D+ and K/aD cells were labeled by a two hours BrdU pulse (Fig. 7A), indicating these cells were actually proliferating. Notably, K/aD cells displayed a higher proliferation rate compared to K/D+ cells (K/aD 69,5±7,6%; K/D+ 43,4±4,1%; p = 0,012).

Thus, about one fourth of the clustered DCX+ cells do proliferate, and as in the SVZ and DG [32], [30] these dividing neuroblasts are clustered together with cells that do not express DCX and shows a higher proliferation rate.

#### Phenotypic analysis of K/aD and K/D+ cells

To characterize the putative progenitor nature of K/aD and K/D+ cells we performed multiple immunofluorescence labeling for DCX, Ki67 and for: 1) molecules normally expressed by SVZ and DG intermediate neuronal progenitors, such as the glial markers BLBP, SOX2 and SOX9, [30], [29]; 2) the EGF receptor (EGFr), a specific marker of SVZ intermediate progenitors, or type C cells, and 3) the marker of subpallial neurons pan-Dlx [32].

In the CBCM striatum the large majority of K/aD cells were positive for the tested glial markers (BLBP: 91.3±1.5%; Sox2: 98.3±1,0%; SOX9: 93.6±11.2; arrowhead in Fig. 7 B,C and Fig. S3A), while K/D+ cells expressed BLBP in reduced proportions (arrow in Fig. 7C; 57.5±7,9%; F_1,2_ = 52.76; p = 0,015) and SOX2 and SOX9 at lower levels (arrow in Fig. 7C and Fig. S3A). The EGFr was expressed by 94.3±3.2% of K/aD cells, whereas only 8.6±2.7% of K/D+ cells expressed this marker (Fig. 7E). In parallel, 95.1+/−2.4% of K/aD cells and virtually all K/D+ cells were immunopositive for a pan-Dlx antibody (Fig. 7D). Interestingly, individual DCX+ cells were negative for all tested markers except pan-Dlx (asterisk in Fig. 7D and data not shown) while the postmitotic cells of the DCX+ clusters also expressed SOX2 and SOX9 suggesting they were at a more immature stage (Fig. 7C and Fig. S3A).

These data indicate that most of the K/aD cells and a part of the K/D+ cells in the CBCM striatum co-express a set of markers associated with intermediate neuronal progenitors in the adult brain. In particular, the organization and antigenic profile of the K/aD cells closely resemble those of SVZ type C cells. These striatal intermediate progenitor-like cells are specifically associated with clustered DCX+ cells and might account for their local genesis.

### DCX+ neuroblasts in CBCM striatum do not express sets of transcription factors associated with the specification of striatal projection neurons

To investigate the lineage and commitment of striatal neuroblasts in CBCM mice, we analyzed the expression of transcription factors (TFs) associated with the specification of either SVZ neuronal precursors (Sp8 and Pax6; [33], [34], [35]), or striatal interneurons (Nkx2.1 and Lhx6; [36]) and projection neurons (Isl1 and CTIP2; [37]).

Striatal DCX+ or Ki67+ cells were never found to be immunopositive for PAX6, Nkx2.1, Lhx6, nor yet Isl-1 (data not shown). On the other hand, all DCX+ cells co-expressed Sp8 and CTIP2 (Fig. 7F and Fig. S2B,C). Interestingly, K/aD cells expressed Sp8 and some of them were also immunopositive for CTIP2 (Fig. 7F; Fig. S2B). Thus, striatal DCX+ neuroblasts and part of the K/aD cells share the expression of Sp8 and CTIP2 suggesting they belong to the same lineage. Notably, this combination of TFs is not associated with the specification of any known striatal neurons during development.

### Survival and differentiation of DCX+ neuroblasts are severely impaired in the CBCM striatum

**Figure 8:**
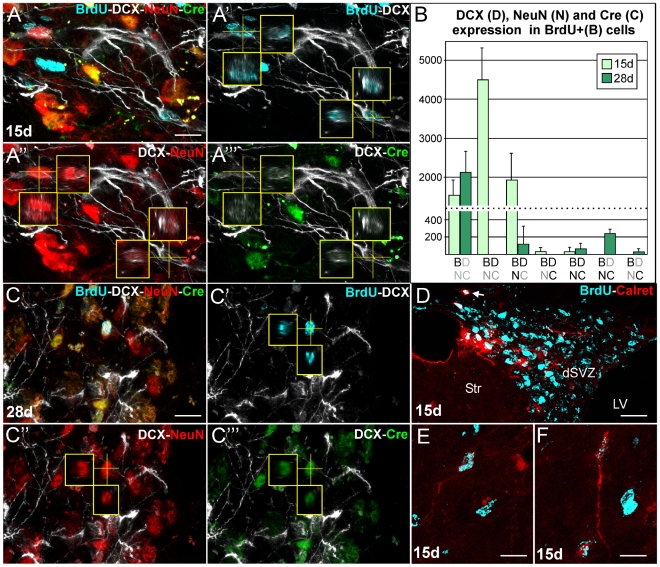
Time course of differentiation and phenotypic analysis of newly generated neuronal cells in the striatum of CBCM mice. **A-A‴ and C-C‴**) Z projections of confocal stacks taken out of 25 µm thick sections simultaneously labeled for BrdU (cyan), DCX (white), NeuN (red) and Cre (green). Small panels show reslices of the stack along the planes indicated by the yellow lines. In A-A‴ two BrdU positive cellst 15 days after the first of 5 BrdU injections labeled for DCX and NeuN but not Cre. In C-C‴ one BrdU positive cell expresses NeuN and Cre but not DCX at 28 days survival time. **B**) Estimates of BrdU (B), DCX (D), NeuN (N) or Cre (C) immunolabeled cells at 15 and 28 days survival times. Different combinations of these antigens are indicated on the x axis with black letters. **D–F**) Images of Calretinin and BrdU labeled sections cut at the level of LV and striatum at 15 d survival time. Note that BrdU positive nuclei accumulate in the dSVZ, some of which express calretinin (arrow). In E and F, two calretinin/BrdU positive cells in the dorsolateral striatum are shown. Scale bars 10 µm in A,C,E,F; 10 µm in D.

To investigate the fate of newly generated cells in the CBCM striatum we injected animals with BrdU for five consecutive days (1 injection/day; 50 mg/Kg) and analyzed the phenotype of BrdU+ cells 15 d (n = 3) and 28 d (n = 3) after the first injection. Simultaneous labeling for BrdU, the immature neuronal marker DCX, the mature neuronal markers NeuN and CaMKII-Cre (Fig. 8A–C) showed that between 15 d and 28 d after BrdU administration, the number of the BrdU+ cells expressing at least one of the tested neuronal markers had greatly decreased (6458±457 cells at 15 d vs 457±220 cells at 35 d; F_1,2_ = 77,77; p = 0,001). At 15 d all BrdU+ cells in the neuronal lineage expressed DCX and 29.5±8% of them were co-labeled for NeuN, whereas only rare cells expressed CaMKII-Cre (Fig. 8B). At 28 d we estimated that 250±46.1 BrdU+ cells/hemisphere expressed CaMKII-Cre, NeuN but not DCX, indicating these cells had reached a relatively mature stage of differentiation. These few mature neurons represented only about 3.8% of the neuroblast population at 15 d.

By coupling BrdU with markers of striatal projection neurons (DARP-32 and FOXP-1), interneurons (Parvalbumin, Somatostatin, Calretinin) and dopaminergic cells (TH), at both 15 d and 28 d survival, only rare double labeled BrdU/Calretinin cells were found (Fig. 8 E,F). BrdU/Calretinin double positive cells were also observed in the dSVZ (Fig. 8D), a region described as a source of such cells in stroke lesioned striatum [10], [12].

Overall, these data indicate that a great majority of newly generated neurons in the striatum of CBCM mice die either before or immediately after expressing the CaMKII-Cre failing to replace the medium spiny neurons.

### Differentiation of transplanted embryonic striatal precursors is not impaired in the CBCM striatum

**Figure 9:**
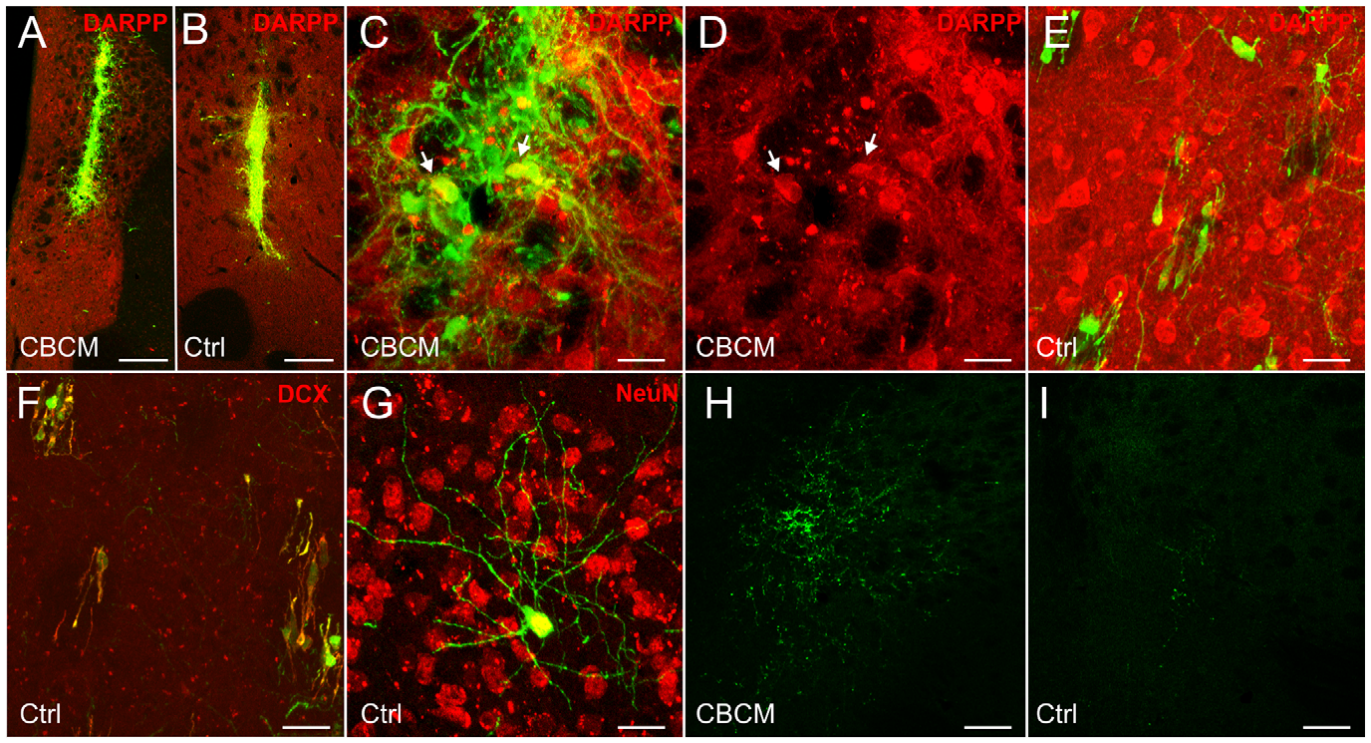
Intrastriatal transplantation of E15 LGE striatal precursors. **A,B**) Coronal sections labelled for EGFP (green) and DARPP-32 (red) at the level of the transplantation site in CBCM (A) and control (B) mice. **C-C′**) Clustered EGFP and DARPP-32 positive medium spiny neurons (arrows) in a transplanted CBCM mouse. **D**) EGFP positive (green) DARPP-32 negative (red) cells in the lateral striatum at a considerable distance from the transplantation site in one control animal. **E**) EGFP+ cells (green) expressing DCX (red). **F**) One differentiated non-spiny EGFP+/NeuN+ cell in one control animal located far from the transplantation site. **G,H**) A higher density of EGFP+ fibers can be observed in CBCM (G) than in control (H) globus pallidus. Scale Bars: 200 µm in A,B; 20 µm in C–H.

Survival and differentiation failure of newborn neurons are common features of both pathologic and physiologic models of striatal neurogenesis [2], [9]. This could be due to a combination of cell autonomous mechanisms and environmental cues. In order to investigate whether the CBCM striatum is permissive for the survival and differentiation of correctly specified cells, we transplanted striatal precursors derived from the lateral ganglionic eminence (LGE) of E15 [38] EGFP+ mice into the striatum of adult CBCM and control mice. At 21 days after transplantation, numerous EGFP+ cells were found in the striatum of both CBCM and control mice (Fig. 9 A–G). Among these cells, many were projection neurons, identifiable for the high spine density and DARPP-32 expression (Fig. 9 C,D). Accordingly, several EGFP+ axons travelled within the internal capsule and reached the globus pallidus (Fig. 9 H,I). In this latter region, axonal fibers were observed to arborize more extensively in CBCM mice than in controls (Fig. 9 H,I).

It is to be noted that in both control and CBCM mice numerous transplanted cells expressing NeuN or DCX dispersed widely from the injection site into the striatum and neocortex (Fig. 9 F,G and data not shown). By contrast, EGFP+/DARPP-32+ projection neurons were mostly restricted to the transplantation site (Fig. 9A–E). These results indicate that the striatal environment, both in CBCM and control mice, is permissive for differentiation and survival but not migration of striatal projection neuron precursors. Moreover, these data also suggest the differentiation failure of endogenous neuroblasts in CBCM striatum is mostly due to cell autonomous factors.

## Discussion

Several studies have analyzed the induction of neurogenesis in non-neurogenic regions of the adult brain following acute injuries [7]. On the other hand, the neurogenic response to a slow progressive neurodegeneration has been poorly investigated. In this study we examined induced neurogenesis in the striatum of a mouse model of slow progressive neuronal degeneration: the CREB1^Camkcre4^CREM^−/−^ double transgenic mice (CBCM; [16]). The analyses were performed at advanced stages of neurodegeneration (6–8 month old animals), in which the volume of the striatum was reduced by about 70%.

### Origin of the striatal neuroblasts

#### SVZ contribution

In CBCM mice, we demonstrated that SVZ neuroblasts migrate towards the degenerated striatum. These cells reach the CBCM striatum through a specific pathway that runs along the boundary between the corpus callosum and the striatum, or dPSB. This region appears as a lateral extension of the dSVZ. Similarly to the RMS-OB migratory route [39], SVZ-derived neuroblasts initially migrate organized in chains within the dPSB and then detach from the chains to enter the mature striatal parenchyma as individual cells. In contrast to our results in CBCM mice, *in vitro slice* cultures of the striatum shortly after acute ischemic injury, showed that SVZ neuroblasts enter the striatum from the whole periventricular SVZ, often organized as chains [40], [41]. Moreover, differently from acute striatal lesions [2], [6], [3] the SVZ showed normal proliferative levels in CBCM mice. These differences further support the fact that acute and chronic degenerative conditions are associated with tissue responses that differentially modulate distinct aspects of adult neurogenesis [14], [15].

#### Local neuronal genesis contribution

Although our analyses in CBCM mice indicated that SVZ neuroblasts migrate individually within the striatum, about half of the striatal DCX+ cells were organized in clusters. Since more than 60% of the clustered DCX+ cells were generated within five days, our data suggest these cells might not have migrated from the SVZ. In line with this hypothesis, we found strong evidence that clustered DCX+ cells are generated within the striatal parenchyma. Indeed, about one fourth of these cells expressed the endogenous marker of cell proliferation Ki67 (K/D+ cells). Proliferative activity of the K/D+ cells was further demonstrated by two hour BrdU pulse labeling and by the observation of mitotic figures.

Parenchymal generation of neuroblasts had previously been demonstrated in the normal rabbit striatum, where similar clustered proliferating neuroblasts were observed [9]. This finding was further supported by *in vitro* experiments in which BrdU labeled neuroblasts migrated out of striatal explants obtained from BrdU pulse labeled animals [9]. Unfortunately, in CBCM mice we could not obtain *in vitro* migration of neuroblasts neither from SVZ nor striatal explants, which is likely due to the poor viability of such explants in aged mice (Luzzati et al. unpublished observation).

**Figure 10:**
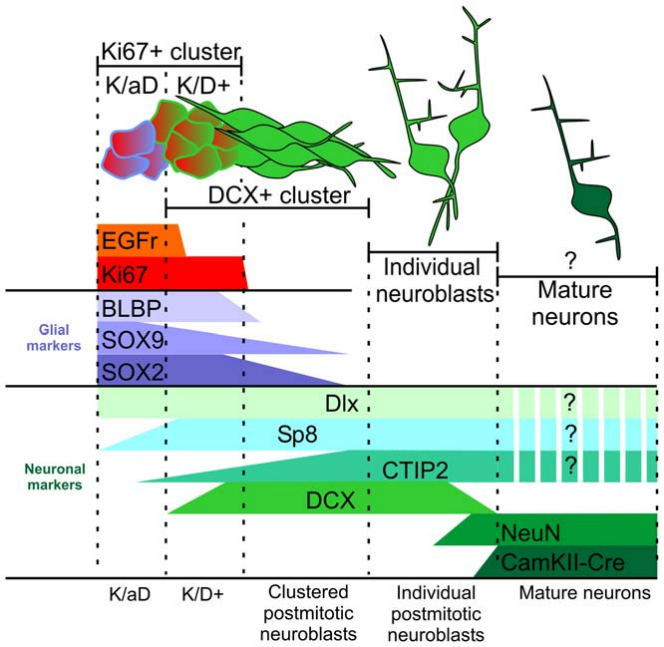
Proposed developmental progression of local parenchymal progenitors in CBCM striatum. In this model highly proliferating Ki67+ clusters give rise to DCX+ neuroblasts initially organized in clusters and then as individual cells. These cells eventually differentiate into DCX−, NeuN+, CamKIIa-Cre+ mature neurons. The Ki67+ cluster are made by the K/aD cells that express EGFr but not DCX and K/D+ cells that express DCX but not the EGFr, both cell types express SOX2, BLBP, SOX9, Dll and Sp8. Postmitotic neuroblasts downregulate BLBP but maintain SOX2 and SOX9 expression until they are part of the DCX+ clusters. The entire lineage is characterized by Sp8 and Dll expression. CTIP2 is also expressed by all DCX+ cells but only a few K/aD cells. A question mark indicates that the expression of Sp8, CTIP2 and Dll has not been determined in DCX negative newly generated striatal neurons.

Since SVZ migrating neuroblasts are able to divide after their genesis [42], [43], and that cell division would likely dilute CTG labeling, we cannot exclude an SVZ origin of K/D+ cells. Nonetheless, considerable evidence supports the hypothesis that these cells were produced in the context of a more specialized neurogenic system. Indeed, as in the SVZ, DG and rabbit striatum [24], [9], [29], proliferating neuroblasts of CBCM striatum were specifically associated with Ki67+/DCX negative cells (K/aD cells). In contrast to the SVZ neuroblasts migrating to the CBCM striatum, the K/D+ and K/aD cells showed characteristic features of intermediate progenitors including: mitotic activity, clustering, co-expression of markers of both glial (BLBP, SOX2, SOX9) and neuronal lineages (pan-Dlx; Sp8 and in part DCX), expression of the EGFr, and close contact with post-mitotic neuroblasts (Fig. 10) [32], [30], [28], [29]. In particular, the organization and molecular profile of the K/aD cells closely resemble those of SVZ type C cells. The origin of these putative striatal parenchymal neuronal progenitors remains to be determined, however, two main possibilities can be considered: i) they could derive from the SVZ through displacement of type B/C cells, or alternatively ii) they could represent local cells that became neurogenic in response to neurodegeneration. The latter possibility would be consistent with previous *in vitro* studies indicating the presence of neurogenic progenitors in different brain regions including the striatum [44], [45], and with recent *in vivo* evidence showing the activation of local parenchymal progenitors in the neocortex after mild ischemia [8]. Both the migratory capacity of SVZ progenitors and the neurogenic potential of local cells would be of great interest for neural stem cell research, and deserve further investigation.

### Commitment and fate of striatal neuroblasts

In agreement with other pathologic and physiologic models of striatal neurogenesis [2], [6], [9], phenotypic analyses showed that induced neuroblasts in the CBCM striatum did not survive or differentiate into striatal neuronal types, except for a few calretinin expressing cells [9], [46], [10]. This could be due to cell-autonomous rather than environmental factors, because LGE derived embryonic striatal precursors engrafted into the CBCM striatum correctly differentiated into projection neurons.

One possible cell-autonomous factor reducing the survival of endogenous striatal neuroblasts in CBCM mice is CREB deletion, which occurs under the control of CaMKIIa promoter [16]. However, in these animals CaMKIIa drives Cre expression at late neuronal differentiation stages, leading to normal development [16], and only 25% reduction in adult-generated OB interneuron survival [27]. The dramatic loss of striatal newborn neurons occurring in CBCM mice suggests that other cell-autonomous factors can affect the survival of these cells. As in the post-stroke rat striatum [10], striatal neuroblasts in CBCM mice do not express a set of TFs specifically involved in the differentiation of medium spiny neurons, but express Sp8, which is also found in SVZ cells fated to become OB interneurons and striatal calretinin interneurons [35], [12]. Interestingly, in the normal post-natal guinea pig striatum we recently observed a population of neuroblasts having a transient existence and showing Sp8+ expression [47]; Luzzati F unpublished results]. These cells were in contact with the ventral PSB, and similar to the neuroblasts of the dPSB connected groups of CBCM mice, they were closely associated with internal capsule fiber bundles [47], (Luzzati F unpublished results). These observations are consistent with the possibility, so far not considered, that striatal neurodegeneration may stimulate the production of a transient neuronal cell type. that might be involved in some forms of reconfiguration of the incoming cortical and/or thalamic inputs.

### Concluding remarks

The mature brain parenchyma has been classically considered non permissive for neuronal progenitor activity. This idea comes mostly from the observations that transplantation of stem/progenitor cells into the mature brain parenchyma mainly gives rise to glial cells [48], [49], [50]. Transplantation of SVZ neurospheres in the striatum of CBCM mice yielded similar results (Luzzati et al. unpublished results). Nonetheless, our data in CBCM mice and in normal rabbits, together with recent studies in the neocortex [9], [8] indicate that parenchymal neuronal production can occur in specific circumstances. A deeper understanding of the biology of the parenchymal neuronal progenitors could feed the development of cell replacement/restorative therapies based on local neuronal production. Future studies should also address whether, as for SVZ neuronal progenitors [51], [52], activation of ectopic neurogenic progenitors, either derived from migrating SVZ progenitors or local cells, can give rise to brain tumors.

## Supporting Information

Figure S1 **3D reconstruction study: relationships of the striatal DCX+ clusters with the SVZ and dPSB in control and mutant animals.** Lateral (at top) and bottom views (at bottom) of the 3D models shown in fig. 2. Control on the left, CBCM on the right (voxel size: 0,7×0,7×40 µm). The X (gray), Y (dark) and Z (cyan) axes are indicated for each view. The graduate scale indicates the section number.
(TIF)

Figure S2 **3D reconstruction of six representative internal capsule fiber bundles (voxel size: 0,7×0,7×40 µm).** Frontal (on the left) and lateral views (on the right) of a 3D model obtained from sections #130 to 110 of the same material of Fig. 2 and 3, showing the corpus callosum (gray), the lateral ventricle (yellow) the SVZ (dPSB (green) and six representative internal capsule fibre bundles (blue) The graduate scale indicates the section number.
(TIF)

Figure S3 **Expression of SOX9 and CTIP2 in K/D+, K/aD and Ki67− DCX+ cells.** A–C) Z projection of confocal stacks taken out of 25 µm thick sections triple labelled for Ki67 (green), DCX (white) and in red: SOX9 (A-A‴) and CTIP2 (B-B‴; C-C‴). Insets in A,B are single confocal planes in which the labelling of K/D+ (arrows), K/aD (arrowheads) and postmitotic DCX+ cells can be better appreciated. In C arrows indicate two DCX positive individual cells expressing CTIP2 but not Ki67. Scale Bars 10 µm.
(TIF)

Video S1 **LSCM serial section reconstruction of two dPSB connected groups.** The first part of this video shows a 360° rotation, beginning with a posterior view, of the maximum intensity projection (MIP) volume rendering of an LSCM serial section reconstruction (sections #122–127; voxel size 0,13×0,13×1 µm) encompassing a cuboid volume of the dorso-lateral striatum (base: 240×240 µm). In this volume the lateral part of the dPSB and part of two *dPSB connected groups* (blue and cyan of Fig. 3D) are shown. In the second part of the video tracings of the dPSB chains and of the two *dPSB connected groups*, obtained with neuronstudio, are shown. Note the complete absence of clustered DCX+ cells in these groups.
(MOV)

## References

[1] Kriegstein A, Alvarez-Buylla A (2009). The glial nature of embryonic and adult neural stem cells.. Annu Rev Neurosci.

[2] Arvidsson A, Collin T, Kirik D, Kokaia Z, Lindvall O (2002). Neuronal replacement from endogenous precursors in the adult brain after stroke.. Nat Med.

[3] Parent JM, Vexler ZS, Gong C, Derugin N, Ferriero DM (2002). Rat forebrain neurogenesis and striatal neuron replacement after focal stroke.. Ann Neurol.

[4] Nakatomi H, Kuriu T, Okabe S, Yamamoto S, Hatano O (2002). Regeneration of hippocampal pyramidal neurons after ischemic brain injury by recruitment of endogenous neural progenitors.. Cell.

[5] Magavi SS, Leavitt BR, Macklis JD (2000). Induction of neurogenesis in the neocortex of adult mice.. Nature.

[6] Collin T, Arvidsson A, Kokaia Z, Lindvall O (2005). Quantitative analysis of the generation of different striatal neuronal subtypes in the adult brain following excitotoxic injury.. Exp Neurol.

[7] Kernie SG, Parent JM (2010). Forebrain neurogenesis after focal Ischemic and traumatic brain injury.. Neurobiol Dis.

[8] Ohira K, Furuta T, Hioki H, Nakamura KC, Kuramoto E (2010). Ischemia-induced neurogenesis of neocortical layer 1 progenitor cells.. Nat Neurosci.

[9] Luzzati F, De Marchis S, Fasolo A, Peretto P (2006). Neurogenesis in the caudate nucleus of the adult rabbit.. J Neurosci.

[10] Liu F, You Y, Li X, Ma T, Nie Y (2009). Brain injury does not alter the intrinsic differentiation potential of adult neuroblasts.. J Neurosci.

[11] Yamashita T, Ninomiya M, Hernandez Acosta P, Garcia-Verdugo JM (2006). Subventricular zone-derived neuroblasts migrate and differentiate into mature neurons in the post-stroke adult striatum.. J Neurosci.

[12] Wei B, Nie Y, Li X, Wang C, Ma T (2011). Emx1-expressing neural stem cells in the subventricular zone give rise to new interneurons in the ischemic injured striatum.. Eur J Neurosci.

[13] Batista CM, Kippin TE, Willaime-Morawek S, Shimabukuro MK, Akamatsu W (2006). A progressive and cell non-autonomous increase in striatal neural stem cells in the Huntington's disease R6/2 mouse.. J Neurosci.

[14] Buffo A, Rolando C, Ceruti S (2010). Astrocytes in the damaged brain: molecular and cellular insights into their reactive response and healing potential.. Biochem Pharmacol.

[15] Whitney NP, Eidem TM, Peng H, Huang Y, Zheng JC (2009). Inflammation mediates varying effects in neurogenesis: relevance to the pathogenesis of brain injury and neurodegenerative disorders.. J Neurochem.

[16] Mantamadiotis T, Lemberger T, Bleckmann SC, Kern H, Kretz O (2002). Disruption of CREB function in brain leads to neurodegeneration.. Nat Genet.

[17] Cong SY, Pepers BA, Evert BO, Rubinsztein DC, Roos RA (2005). Mutant huntingtin represses CBP, but not p300, by binding and protein degradation.. Mol Cell Neurosci.

[18] Jiang H, Poirier MA, Liang Y, Pei Z, Weiskittel CE (2006). Depletion of CBP is directly linked with cellular toxicity caused by mutant huntingtin.. Neurobiol Dis.

[19] Carletti B, Grimaldi P, Magrassi L, Rossi F (2004). Engraftment and differentiation of neocortical progenitor cells transplanted to the embryonic brain in utero.. J Neurocytol.

[20] De Marchis S, Bovetti S, Carletti B, Hsieh YC, Garzotto D (2007). Generation of distinct types of periglomerular olfactory bulb interneurons during development and in adult mice: implication for intrinsic properties of the subventricular zone progenitor population.. J Neurosci.

[21] Rodriguez A, Ehlenberger DB, Hof PR, Wearne SL (2006). Rayburst sampling, an algorithm for automated three-dimensional shape analysis from laser scanning microscopy images.. Nat Protoc.

[22] Peng H, Ruan Z, Long F, Simpson JH, Myers EW (2010). V3D enables real-time 3D visualization and quantitative analysis of large-scale biological image data sets.. Nat Biotechnol.

[23] Kempermann G, Kuhn HG, Gage FH (1997). Genetic influence on neurogenesis in the dentate gyrus of adult mice.. Proc Natl Acad Sci U S A.

[24] Kronenberg G, Reuter K, Steiner B, Brandt MD, Jessberger S (2003). Subpopulations of proliferating cells of the adult hippocampus respond differently to physiologic neurogenic stimuli.. J Comp Neurol.

[25] Gordon RJ, Tattersfield AS, Vazey EM, Kells AP, McGregor AL (2007). Temporal profile of subventricular zone progenitor cell migration following quinolinic acid-induced striatal cell loss.. Neuroscience.

[26] De Marchis S, Fasolo A, Shipley M, Puche A (2001). Unique neuronal tracers show migration and differentiation of SVZ progenitors in organotypic slices.. J Neurobiol.

[27] Giachino C, De Marchis S, Giampietro C, Parlato R, Perroteau I (2005). cAMP response element-binding protein regulates differentiation and survival of newborn neurons in the olfactory bulb.. J Neurosci.

[28] Suh H, Consiglio A, Ray J, Sawai T, D'Amour KA (2007). In vivo fate analysis reveals the multipotent and self-renewal capacities of Sox2+ neural stem cells in the adult hippocampus.. Cell Stem Cell.

[29] Cheng LC, Pastrana E, Tavazoie M, Doetsch F (2009). miR-124 regulates adult neurogenesis in the subventricular zone stem cell niche.. Nat Neurosci.

[30] Steiner B, Klempin F, Wang L, Kott M, Kettenmann H (2006). Type-2 cells as link between glial and neuronal lineage in adult hippocampal neurogenesis.. Glia.

[31] Scholzen T, Gerdes J (2000). The Ki-67 protein: from the known and the unknown.. J Cell Physiol.

[32] Doetsch F, Petreanu L, Caille I, Garcia-Verdugo JM, Alvarez-Buylla A (2002). EGF converts transit-amplifying neurogenic precursors in the adult brain into multipotent stem cells.. Neuron.

[33] Hack MA, Saghatelyan A, de Chevigny A, Pfeifer A, Ashery-Padan R (2005). Neuronal fate determinants of adult olfactory bulb neurogenesis.. Nat Neurosci.

[34] Kohwi M, Osumi N, Rubenstein JL, Alvarez-Buylla A (2005). Pax6 is required for making specific subpopulations of granule and periglomerular neurons in the olfactory bulb.. J Neurosci.

[35] Waclaw RR, Allen ZJ, Bell SM, Erdelyi F, Szabo G (2006). The zinc finger transcription factor Sp8 regulates the generation and diversity of olfactory bulb interneurons.. Neuron.

[36] Marin O, Anderson SA, Rubenstein JL (2000). Origin and molecular specification of striatal interneurons.. J Neurosci.

[37] Arlotta P, Molyneaux BJ, Jabaudon D, Yoshida Y, Macklis JD (2008). Ctip2 controls the differentiation of medium spiny neurons and the establishment of the cellular architecture of the striatum.. J Neurosci.

[38] Mason HA, Rakowiecki SM, Raftopoulou M, Nery S, Huang Y (2005). Notch signaling coordinates the patterning of striatal compartments.. Development.

[39] Whitman MC, Greer CA (2009). Adult neurogenesis and the olfactory system.. Prog Neurobiol.

[40] Zhang RL, Chopp M, Gregg SR, Toh Y, Roberts C (2009). Patterns and dynamics of subventricular zone neuroblast migration in the ischemic striatum of the adult mouse.. J Cereb Blood Flow Metab.

[41] Kojima T, Hirota Y, Ema M, Takahashi S, Miyoshi I (2010). Subventricular zone-derived neural progenitor cells migrate along a blood vessel scaffold toward the post-stroke striatum.. Stem Cells.

[42] Zhang RL, LeTourneau Y, Gregg SR, Wang Y, Toh Y (2007). Neuroblast division during migration toward the ischemic striatum: a study of dynamic migratory and proliferative characteristics of neuroblasts from the subventricular zone.. J Neurosci.

[43] Menezes JR, Smith CM, Nelson KC, Luskin MB (1995). The division of neuronal progenitor cells during migration in the neonatal mammalian forebrain.. Mol Cell Neurosci.

[44] Buffo A, Rite I, Tripathi P, Lepier A, Colak D (2008). Origin and progeny of reactive gliosis: A source of multipotent cells in the injured brain.. Proc Natl Acad Sci U S A.

[45] Palmer TD, Markakis EA, Willhoite AR, Safar F, Gage FH (1999). Fibroblast growth factor-2 activates a latent neurogenic program in neural stem cells from diverse regions of the adult CNS.. J Neurosci.

[46] Dayer AG, Cleaver KM, Abouantoun T, Cameron HA (2005). New GABAergic interneurons in the adult neocortex and striatum are generated from different precursors.. J Cell Biol.

[47] Luzzati F, Fasolo A, Peretto P (2011). Combining confocal laser scanning microscopy with serial section reconstruction in the study of adult neurogenesis.. Front Neurosci.

[48] Gage FH (2000). Mammalian neural stem cells.. Science.

[49] Eriksson C, Bjorklund A, Wictorin K (2003). Neuronal differentiation following transplantation of expanded mouse neurosphere cultures derived from different embryonic forebrain regions.. Exp Neurol.

[50] Burns TC, Verfaillie CM, Low WC (2009). Stem cells for ischemic brain injury: a critical review.. J Comp Neurol.

[51] Liu HK, Wang Y, Belz T, Bock D, Takacs A (2010). The nuclear receptor tailless induces long-term neural stem cell expansion and brain tumor initiation.. Genes Dev.

[52] Alcantara Llaguno S, Chen J, Kwon CH, Jackson EL, Li Y, Burns DK (2009). Malignant astrocytomas originate from neural stem/progenitor cells in a somatic tumor suppressor mouse model.. Cancer Cell.

[53] Panganiban G, Sebring A, Nagy L, Carroll S (1995). The development of crustacean limbs and the evolution of arthropods.. Science.

[54] Grigoriou M, Tucker AS, Sharpe PT, Pachnis V (1998). Expression and regulation of Lhx6 and Lhx7, a novel subfamily of LIM homeodomain encoding genes, suggests a role in mammalian head development.. Development.

