# Supplementary figures and images for: New Striatal Neurons in a Mouse Model of Progressive Striatal Degeneration Are Generated in both the Subventricular Zone and the Striatal Parenchyma
Source: PLoS One. 2011 Sep 30;6(9):e25088. doi: 10.1371/journal.pone.0025088 (PMC3184103; doi:10.1371/journal.pone.0025088)

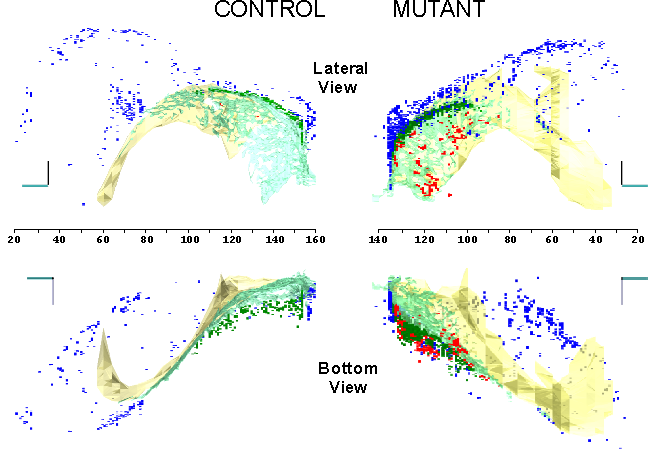

Supplement: Figure S1 — 3D reconstruction study: relationships of the striatal DCX+ clusters with the SVZ and dPSB in control and mutant animals. Lateral (at top) and bottom views (at bottom) of the 3D models shown in fig. 2. Control on the left, CBCM on the right (voxel size: 0,7×0,7×40 µm). The X (gray), Y (dark) and Z (cyan) axes are indicated for each view. The graduate scale indicates the section number. (TIF) [file pone.0025088.s001.tif]

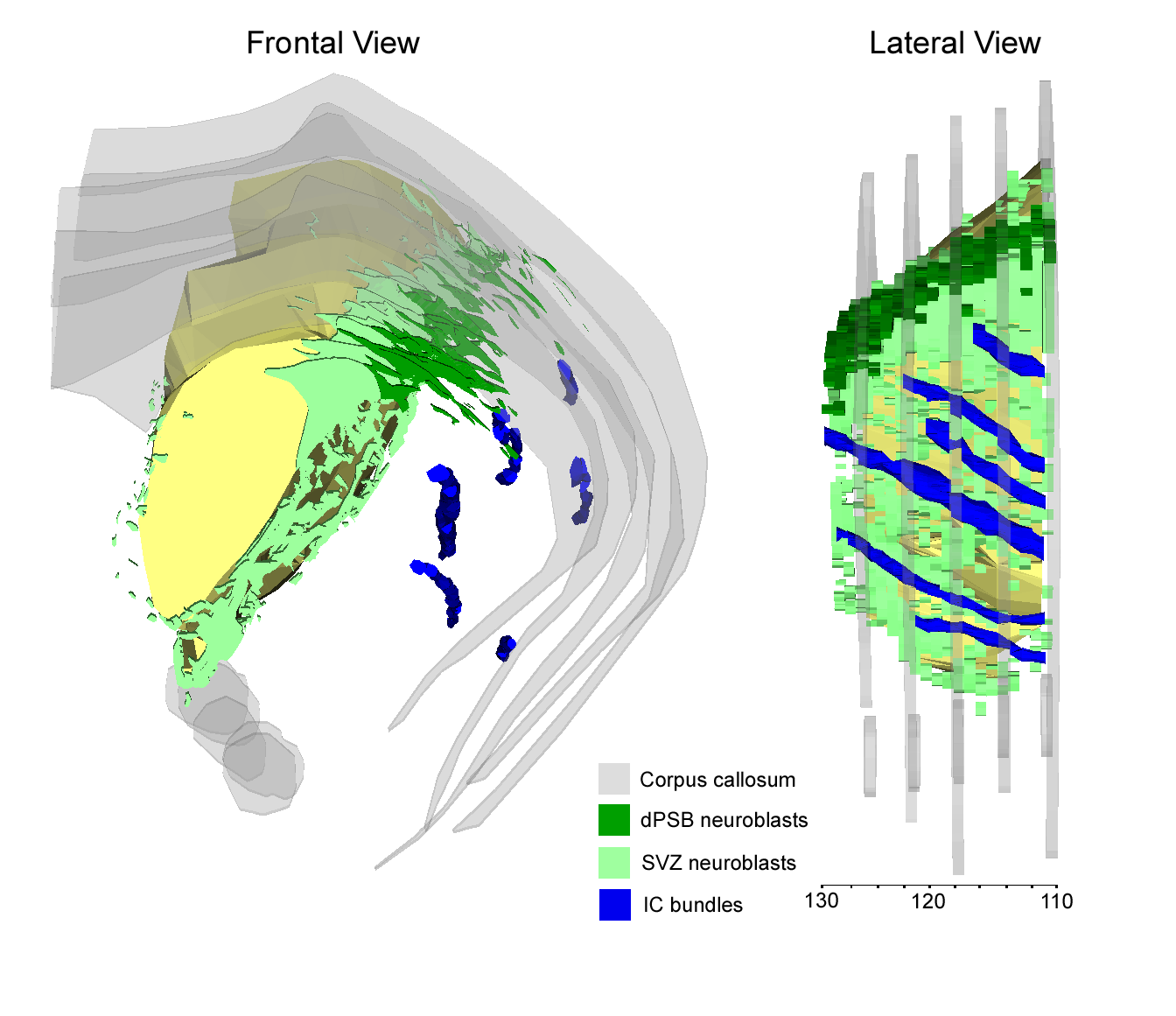

Supplement: Figure S2 — 3D reconstruction of six representative internal capsule fiber bundles (voxel size: 0,7×0,7×40 µm). Frontal (on the left) and lateral views (on the right) of a 3D model obtained from sections #130 to 110 of the same material of Fig. 2 and 3, showing the corpus callosum (gray), the lateral ventricle (yellow) the SVZ (dPSB (green) and six representative internal capsule fibre bundles (blue) The graduate scale indicates the section number. (TIF) [file pone.0025088.s002.tif]

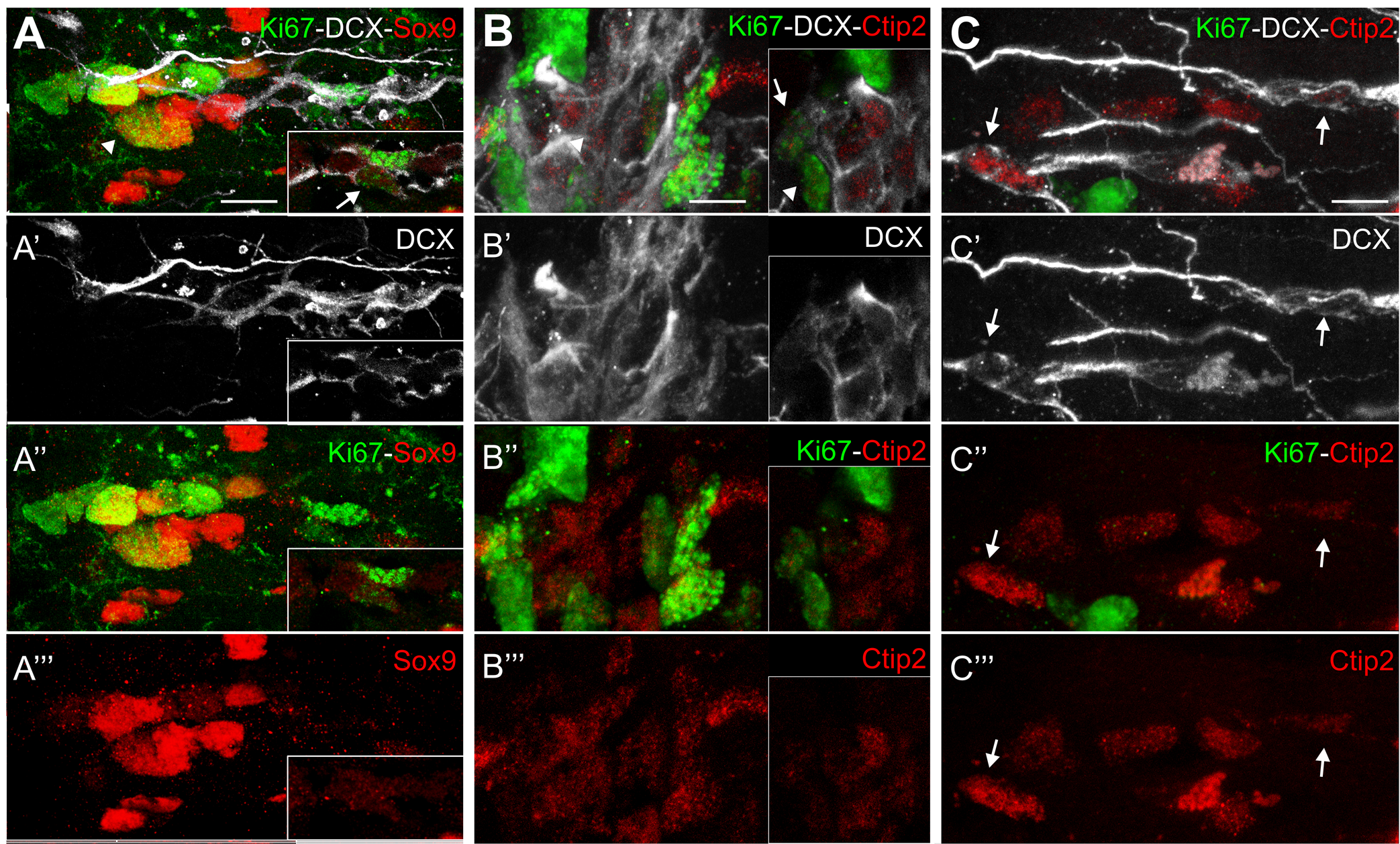

Supplement: Figure S3 — Expression of SOX9 and CTIP2 in K/D+, K/aD and Ki67− DCX+ cells. A–C) Z projection of confocal stacks taken out of 25 µm thick sections triple labelled for Ki67 (green), DCX (white) and in red: SOX9 (A-A‴) and CTIP2 (B-B‴; C-C‴). Insets in A,B are single confocal planes in which the labelling of K/D+ (arrows), K/aD (arrowheads) and postmitotic DCX+ cells can be better appreciated. In C arrows indicate two DCX positive individual cells expressing CTIP2 but not Ki67. Scale Bars 10 µm. (TIF) [file pone.0025088.s003.tif]
